# The Spin–Orbit Effect on the Electronic Structures, Refractive Indices, and Birefringence of X_2_PO_4_I (X = Pb, Sn, Ba and Sr): A First-Principles Investigation

**DOI:** 10.3390/nano14070617

**Published:** 2024-04-01

**Authors:** Xudong Leng, Mei Hu, Qun Jing, Haiming Duan, Henglei Chen, Xiuhua Cui

**Affiliations:** Xinjiang Key Laboratory of Solid State Physics and Devices, School of Physical Science and Technology, Xinjiang University, Urumqi 830017, China; chillsir@stu.xju.edu.cn (X.L.); humei09292022@163.com (M.H.); dhm@xju.edu.cn (H.D.); chl@xju.edu.cn (H.C.)

**Keywords:** first principles, spin–orbit coupling, electronic structure, birefringence, lone-pair electrons

## Abstract

Introducing post-transition metal cations is an excellent strategy for enhancing optical properties. This paper focuses on four isomers, namely the X_2_PO_4_I (X = Pb, Sn, Ba, and Sr) series. For the first time, the paper’s attention is paid to the changes in electronic structure, as well as refractive indices and birefringence, with and without the inclusion of spin–orbit effects in this series. The first-principles results show that spin–orbit effects of the 5p and 6p states found in these compounds lead to splitting of the bands, narrowing of the band gap, enhancement of the lone-pair stereochemistry, and enhancement of the refractive indices and birefringence. Moreover, a comparison of the lone-pair electron phosphates, X_2_PO_4_I (X = Pb and Sn), and the isomeric alkaline earth metal phosphates, X_2_PO_4_I (X = Ba and Sr), reveals that changes in the band structure have a greater effect on the enhancement of the birefringence than the slight enhancement of the lone-pair stereochemical activity. This study has important implications for a deeper understanding of the optical properties of crystals and the design of novel optical materials.

## 1. Introduction

Post-transition metal cations, including Pb^2+^, Sn^2+^, Bi^3+^, and Sb^3+^, have a wide variety of applications in electrochemical energy storage [1,2] high-temperature superconductivity [3,4,5], catalysis [5,6,7], gas sensors [8,9,10], birefringent material [11,12,13,14], nonlinear optical crystals [15,16,17,18], etc. By analyzing many existing studies, it was found that the introduction of post-transition metal cations containing stereochemically active lone-pair electrons is a good strategy to enhance optical properties. Evidence for this is found in Pb_2_Ba_3_(BO_3_)_3_Cl [19], KPb_2_(PO_3_)_5_ [20], Rb_2_SbF_3_(NO_3_)_2_ [21], K_2_Sb(P_2_O_7_)F [22], CsSbF_2_SO_4_ [23], Cd_4_BiO(BO_3_)_3_ [24], Bi(IO_3_)F [25], etc. Recently, [SnOX] polyhedra have been successfully introduced into borates and phosphates, and the optical properties were found to be significantly enhanced. Pan et al. reported Sn_2_B_5_O_9_X (X = Cl, Br) [26,27], which comprehensively verified the gain effect of introducing stereochemically active lone-pair electrons from both theoretical and experimental points of view, with significant enhancement of the optical properties as compared to isostructural alkaline-earth metal borates, i.e., M_2_B_5_O_9_X (M = Ca, Sr, Ba, Pb, and Eu; X = Cl, Br, and I) [28]. Subsequently, Sn_2_PO_4_I [29] with great optical anisotropy (≥0.664 @ 546 nm) was obtained in non-π-conjugated phosphates, realizing a breakthrough in inorganic compounds regarding birefringence. However, for the isomeric X_2_PO_4_I (X = Pb, Ba, and Sr) [30] series of Sn_2_PO_4_I, only the structure has been reported, and there is still a gap in the studies on its optical properties.

Notably, for post-transition oxides, especially those containing heavier elements like Pb and Bi, the spin–orbit coupling (SOC) effect is inevitably present, which can have an effect on the electronic structure and optical properties of the compounds. At present, SOC has a wide range of research applications in many fields. For example, in two-dimensional materials, the SOC effect induces phenomena such as band splitting [31] and the spin Hall effect [32]. In the field of nonlinear optics, Narsimha Rao et al. [33]. investigated the effect of the role played by the spin–orbit interaction on the electronic structures and optical properties of CsPbCO_3_F using the first-principles method. The results showed considerable splitting of valence bands (VB) and conduction bands (CB) after the inclusion of spin–orbit interaction, which led to a reduction in the bandgap (from 5.58 eV to 4.45 eV using the Tran-Blaha modified Becke–Johnson method) and enhanced birefringence (from 0.1049 to 0.1057). Recently, Kang and Huang et al. [34] elucidated the unique impact of SOC effects on *α*-BiB_3_O_6_ (BIBO)’s second harmonic generation (SHG) response. Importantly, the SOC effect can significantly improve the accuracy of calculated SHG coefficients of BIBO, which are closer to the experimental values. To the best of our knowledge, most investigations on optical materials have neglected the influence on spin–orbit effects; in particular, there no symmetric study of the electronic structure and optical properties of families of isomers containing different post-transition metals has been conducted. Will the stereochemical activity of lone pairs be affected by the splitting of bands induced by the spin–orbit effect? And would the electronic structures and optical properties of isostructural alkaline-earth metal compounds also be affected by spin–orbit effects like post-transition metal compounds?

Based on these reflections, we focused on the X_2_PO_4_I (X = Pb, Sn, Ba, and Sr) system, which was studied using the first-principles method. X_2_PO_4_I (X = Pb, Sn, Ba, and Sr), which contains both heavier elements and outstanding optical properties, is a system well worth discussing with respect to the spin–orbit effect. However, only Sn_2_PO_4_I [29] has received attention in the field of nonlinear optics, where experimental and theoretical analyses have been reported, so it is necessary to study the SOC effect of X_2_PO_4_I (X = Pb, Sn, Ba, and Sr) on the electronic structure and optical properties. A comparative discussion was carried out by including and excluding the SOC effect for each case. The band gap, as well as the refractive indices and birefringence of the X_2_PO_4_I (X = Pb, Sn, Ba, and Sr) system were observed. In Sn_2_PO_4_I [29] and Pb_2_PO_4_I, it was found that the introduction of the spin–orbit effect results in a decrease of the band gap, an enhancement of the stereochemical activity of lone-pair electrons, and an increase of the birefringence. In order to further investigate whether it is the gain of lone-pair electrons, the electronic structure, refractive indices, and birefringence of X_2_PO_4_I (X = Ba, and Sr) were investigated, with surprising results. It is confirmed that changes in the band structure due to spin–orbit effects play an important role in the changes of refractive indices and birefringence.

## 2. Computational Methods

The electronic structures, refractive indices, and birefringence of X_2_PO_4_I (X = Pb, Sn, Ba, and Sr) were investigated using the density functional theory (DFT) [35] implemented in the PWmat code [36,37]. The calculations were performed using the structures obtained from the ICSD database (Sn_2_PO_4_I [29]: ICSD-113794; Pb_2_PO_4_I [30]: ICSD-427716; Ba_2_PO_4_I [30]: ICSD-427715; Sr_2_PO_4_I [30]: ICSD-427717). The exchange–correlation functional adopts generalized gradient approximation (GGA) [38] of the Perdew–Burke–Ernzerhof (PBE) [39] functional. The plane wave cutoff energy is set to 70 Ry. The following Monkhorst–Pack [40] k-point meshes in the Brillouin zones were chosen: 8 × 8 × 2 (Sn_2_PO_4_I), 4 × 4 × 4 (Pb_2_PO_4_I), 4 × 4 × 4 (Ba_2_PO_4_I), and 4 × 4 × 4 (Sr_2_PO_4_I). Before investigating the electronic structures and optical properties, the cell and atomic coordinates were relaxed using the conjugate gradient (CG) [41] algorithm, and the convergence criterion was set as 0.01 eV/Å for atomic forces and less than 0.01 eV/Natom for lattice stress. The other computational parameters and convergence criteria retained the default values of the PWmat code. The choice of these computational parameters is good enough to ensure accuracy for the present purpose. The combination of relativistic effective core potentials (RECPs) with these emerging DFT techniques has enabled the study of SOC with remarkable accuracy [42]. In addition to the study reported in this paper, the use of the GGA function to probe the influence of CsPbCO_3_F on the SOC effect has yielded reasonable results, as reported by Narsimha Rao [33] et al. Therefore, the use of GGA functions can assess the impact of SOC effects. Due to the discontinuity in the exchange–correlation energy, the band gap is usually underestimated in calculations using the GGA-PBE function. Therefore, to further verify the accuracy of the bandgap results, we added the calculation of the bandgap of Sn_2_PO_4_I that has reported experimental results using the hybrid function HSE06 [43,44,45] with and without the inclusion of SOC effect.

The investigation of the linear relationship properties is based on the dielectric function ε(ω) = $\epsilon_{\infty }^{\left(1\right)}\left(\omega \right)$ + i$\epsilon_{\infty }^{\left(2\right)}\left(\omega \right)$. Utilizing Kramers–Kronig transformation, we deduced the refractive index by computing the imaginary part of the medium, thus providing a vital theoretical underpinning for the study and application of optical materials.

The imaginary part of the dielectric function is expressed as follows:

(1) $$\epsilon_{\infty }^{\left(2\right)}\left(\omega \right)=2\frac{4\pi^{2}}{\Omega }\frac{1}{\omega^{2}}\frac{1}{N_{k}}\underset{n,m,k}{\sum }\left(f_{nk}-f_{mk}\right){\left|\lambda \cdot p_{nm}\right|}^{2}\delta \left(\varepsilon_{mk}-\varepsilon_{nk}-\hbar \omega \right)$$

where ω is the angular frequency of the photon, Ω is the volume of the unit cell, $N_{k}$ is the integration measure in momentum space, $f_{nk}-f_{mk}$ is the difference between the occupation numbers of electrons at band n and momentum k and band m and momentum k, $\varepsilon_{nk}$ is the energy eigenstate of the electron, $p_{nm}$ is the momentum matrix element between bands n and m, and λ is the coupling strength of the light field.

The real part is obtained through K–K transformation as follows:

(2) $$\epsilon_{\infty }^{\left(1\right)}\left(\omega \right)=1+\frac{1}{\pi }\int_{0}^{\infty }\frac{\epsilon_{\infty }^{\left(2\right)}\left(\omega^{\prime }\right)\omega^{\prime }}{\omega^{\prime 2}-\omega^{2}}d\omega^{\prime }$$

The refractive index *n*(*ω*) is expressed as follows:

(3) $$n\left(\omega \right)={\left[\frac{\sqrt{\epsilon_{1}^{2}+\epsilon_{2}^{2}}+\epsilon_{1}^{2}}{2}\right]}^{\frac{1}{2}}$$

## 3. Results and Discussion

### 3.1. The Electronic Structures, Refractive Indices, and Birefringence of Pb_2_PO_4_I

**Figure 1:**
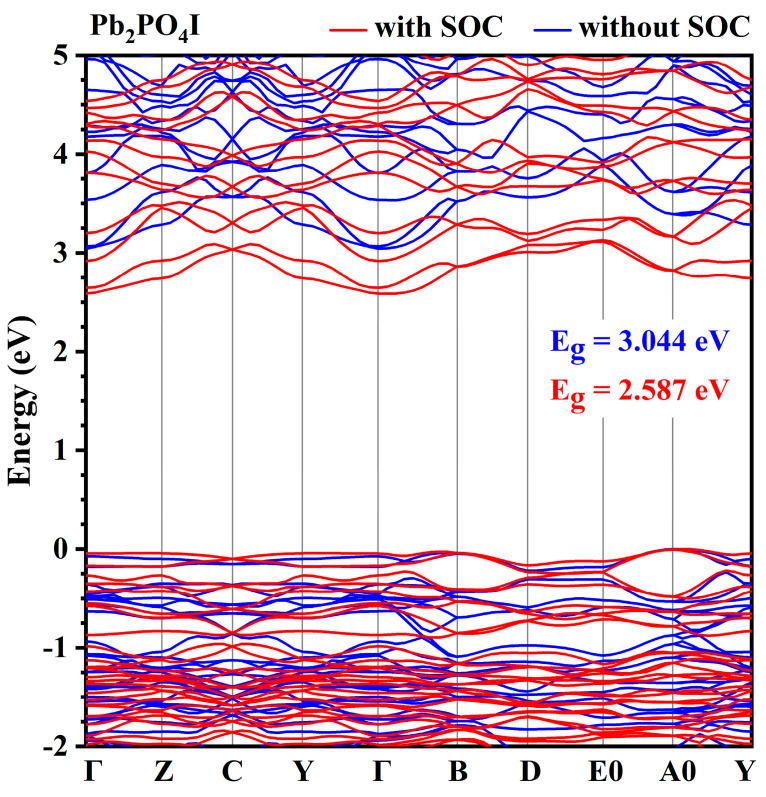
The band structures of Pb_2_PO_4_I along symmetry directions in the first Brillouin zone with (red color) and without (blue color) the inclusion of spin–orbit effect.

Figure 1 presents the obtained band structures of Pb_2_PO_4_I with and without the inclusion of spin–orbit effects. As shown in Figure 1, without the inclusion of spin–orbit effects, Pb_2_PO_4_I is an indirect bandgap semiconductor with the top of the VB located at point A0 and the bottom of the CB located at point Γ, showing a bandgap of 3.044 eV. With the inclusion of the spin–orbit effect, although Pb_2_PO_4_I is still an indirect bandgap semiconductor, the band structure is changed, and a significant splitting situation occurs, with its bandgap decreased to 2.587 eV (shown in Figure 1, Figures S1 and S2). The reduction in bandgap originates from the splitting of the VB and the splitting of the CB after the inclusion of the spin–orbit effect.

**Figure 2:**
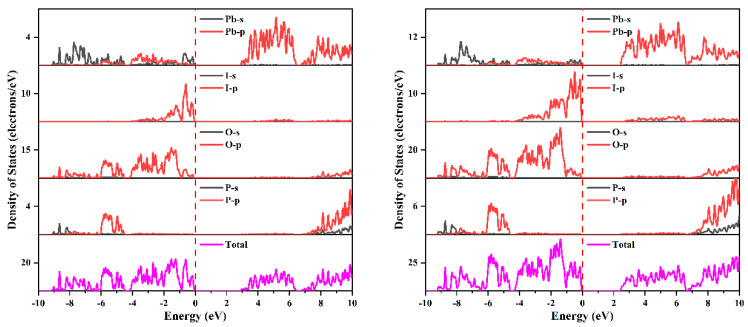
The projected density of states of Pb_2_PO_4_I without (**left panel**) and with (**right panel**) the inclusion of spin–orbit effects.

The splitting of the band may come from the interaction between the spin and the orbit of Pb-p and I-p states. The projected density of states with respect to disregarding and including spin–orbit effects is shown in Figure 2, and the projected band structure of Pb_2_PO_4_I is presented in Figure S2. As shown in Figure 2 and Figure S2, at the top of the VB, there are mainly P-p states, O-p states, I-p states, and Pb-sp states, and at the bottom of the CB, there are mainly Pb-p states. The splitting of the VB near the Fermi level comes from the interaction of spin with I-p states (shown in Figure S2) because a strong spin–orbit effect arises from the interaction between spin and p or d states of heavier elements like 5p states from I and 6p states from Pb. The splitting at the bottom of the CB is thought to be related to the interaction of spin and Pb-p states. The splitting of CB leads to the downshift of CB, resulting in a reduction in bandgap from 3.044 eV to 2.587 eV.

**Figure 3:**
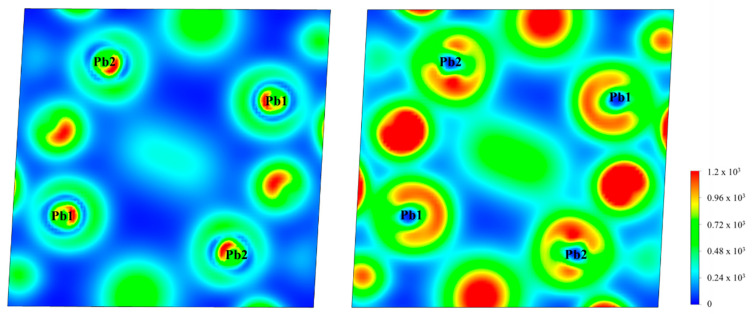
The crystal orbitals in the energy region [−2, 0] eV below the Fermi level of Pb_2_PO_4_I with (**right panel**) and without (**left panel**) the inclusion of spin–orbit effects.

According to the revised model of lone-pair electrons suggested by Walsh et al. [46], the interaction of the antibonding combination of Pb 6s and oxygen p states with unfilled Pb 6p results in asymmetric electron density around Pb(II) cations, which can be confirmed by the crystal orbitals near the Fermi level. Figure 3 presents the crystal orbitals of the energy region [−2, 0] eV below the Fermi level. As expected, one can find asymmetric electron density around Pb(II) cations in Figure 3. Furthermore, compared with the crystal orbitals without the inclusion of spin–orbit effects (shown in the left panel), slightly enhanced stereochemical active lone-pair electrons are found after the inclusion of spin–orbit effects (shown in the right panel of Figure 3). The slightly enhanced stereochemical activity of lone-pair electrons should originate from the splitting the VB after the inclusion of spin–orbit effects (described above).

**Figure 4:**
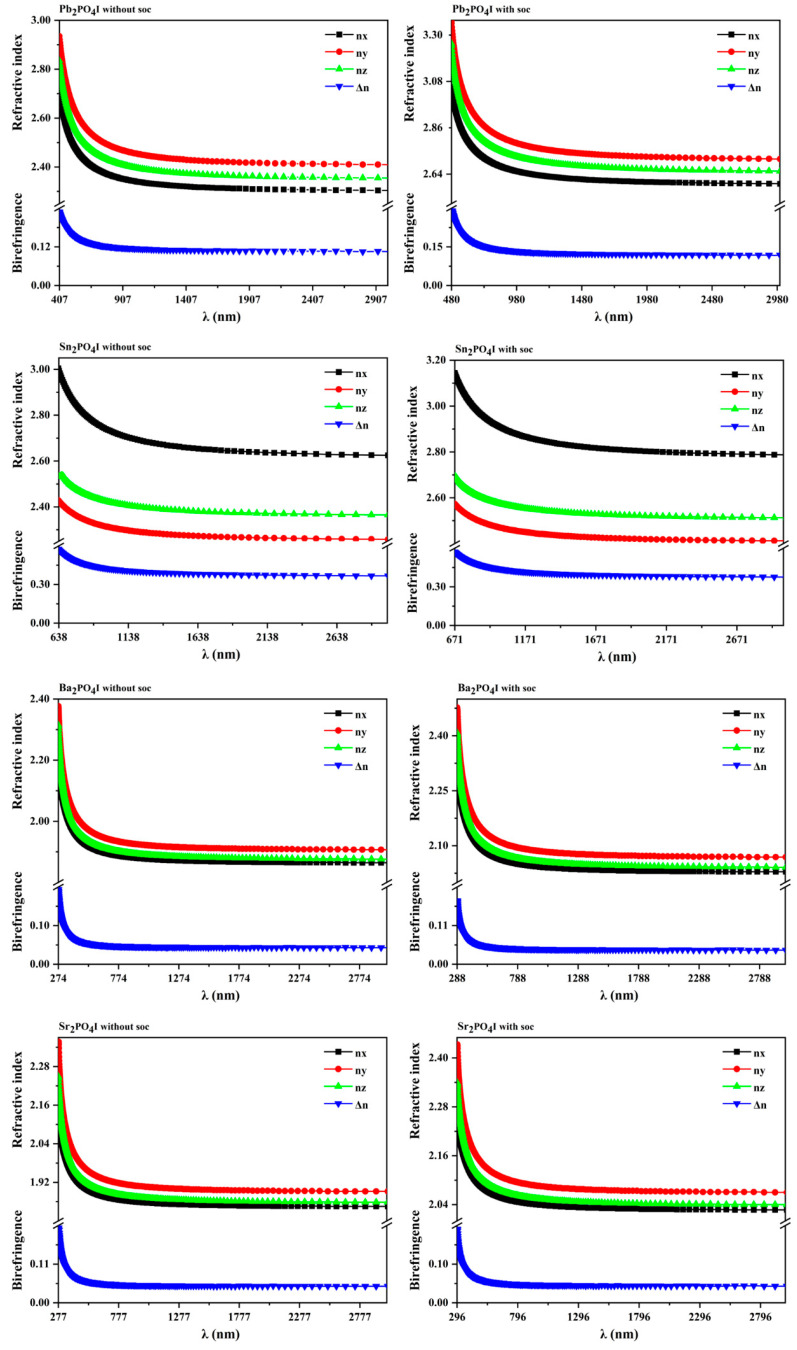
The obtained refractive indices and birefringence of X_2_PO_4_I with and without the inclusion of spin–orbit effects.

Refractive indices and birefringence are also major concerns. Therefore, the refractive indices and birefringence of Pb_2_PO_4_I were further investigated. As shown in Table 1 and Figure 4, without the inclusion of spin–orbit effects, the obtained refractive indices of Pb_2_PO_4_I are 2.393 (n_x_), 2.337 (n_y_), and 2.450 (n_z_) @ 1064 nm, and the birefringence is 0.113 @ 1064 nm. After the inclusion of the spin–orbit effect, the refractive indices of Pb_2_PO_4_I are enhanced to 2.710 (n_x_), 2.643 (n_y_), and 2.774 (n_z_) @ 1064 nm, and the birefringence is enhanced to 0.131 @ 1064 nm. According to the above analysis, the enhanced refractive indices and birefringence have a relation with the reduction in bandgap and enhanced stereochemical activity of lone-pair electrons originating from the splitting of the VB and CB.

### 3.2. The Electronic Structures, Refractive Indices, and Birefringence of Sn_2_PO_4_I

**Figure 5:**
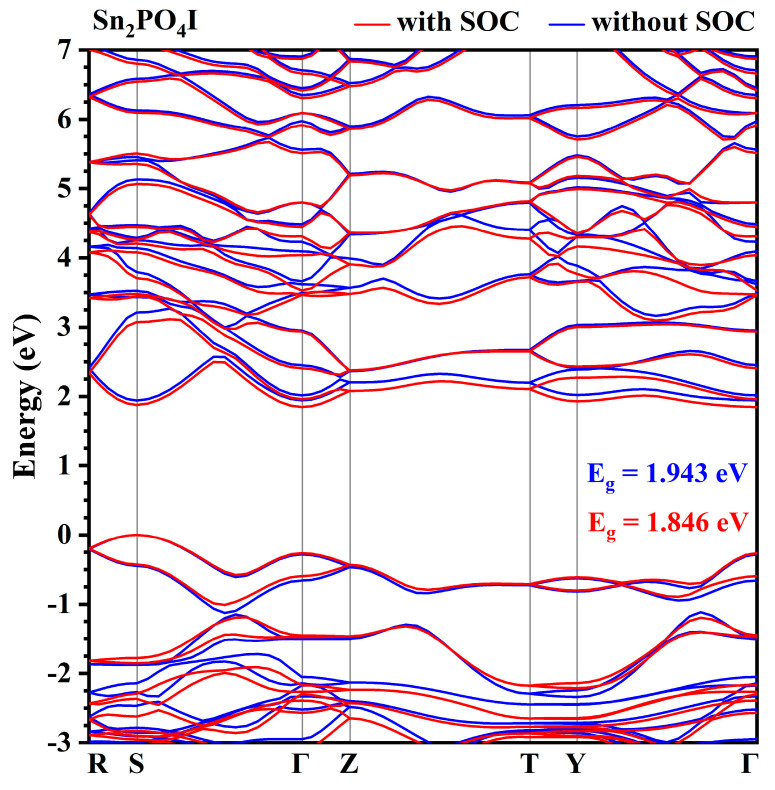
The band structures of Sn_2_PO_4_I along symmetry directions in the first Brillouin zone with (red color) and without (blue color) the inclusion of spin–orbit effects.

Like Pb_2_PO_4_I, the splitting of the VB and CB is also found in Sn_2_PO_4_I (shown in Figure 5, Figures S3 and S4). As shown in Figure 5, without the inclusion of the spin–orbit effect, Sn_2_PO_4_I is a direct band semiconductor with a bandgap of 1.943 eV. Both the top of the VB and the bottom of the CB are located at point S. In addition, HSE06 function calculations show that the HSE06 bandgap of Sn_2_PO_4_I is 2.47 eV without the inclusion of the SOC effect and 2.45 eV with the inclusion of the SOC effect. The HSE06 bandgap with the inclusion of the SOC effect is same as the experimental result (2.45 eV) [29]. The comparative analysis shows that the use of the GGA-PBE function and the HSE06 hybrid function results in the same trend, i.e., both have the band shifted downwards with the inclusion of the spin–orbit effect. The above analyses show that the calculation using the GGA-PBE function for the X_2_PO_4_I (X = Pb, Sn, Ba, and Sr) system is reasonable and does not lead to misinterpretation of the experimental results, in agreement with the experimental results when the SOC effect is taken into account. With the inclusion of the spin–orbit effect, the top of the VB is located at point S, and the bottom of the CB is located at point Γ, which indicates Sn_2_PO_4_I is an indirect bandgap semiconductor with a bandgap of 1.846 eV.

**Figure 6:**
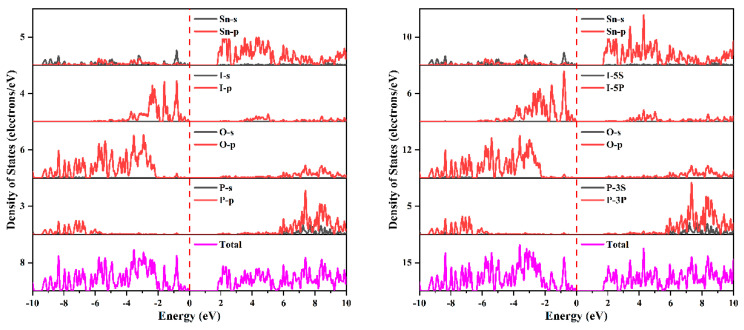
The projected density of states of Sn_2_PO_4_I with and without the inclusion of spin–orbit effects.

Similarly, the splitting of the band may come from the interaction between the spin and the orbit of Sn-p and I-p states. The projected density of states with and without the spin–orbit effect and the projected band structure of Sn_2_PO_4_I are presented in Figure 6 and Figure S4, respectively. As shown in Figure 6 and Figure S4, at the top of the VB, there are mainly P-p states, O-p states, I-p states, and Sn-sp states, and at the bottom of the CB, there are mainly Sn-p states. The splitting of the VB near the Fermi level comes from the interaction of spin with I-p states. The splitting of the bottom of the CB comes from the interaction of spin and Sn-p states. Unlike Pb_2_PO_4_I, the spin–orbit effect of 5p states is smaller than the spin–orbit effect of 6p states, so the reduction in the bandgap of Sn_2_PO_4_I is just 0.097 eV (from 1.943 eV to 1.846 eV with the inclusion of the spin–orbit effect), which is smaller than the 0.457 eV of Pb_2_PO_4_I.

**Figure 7:**
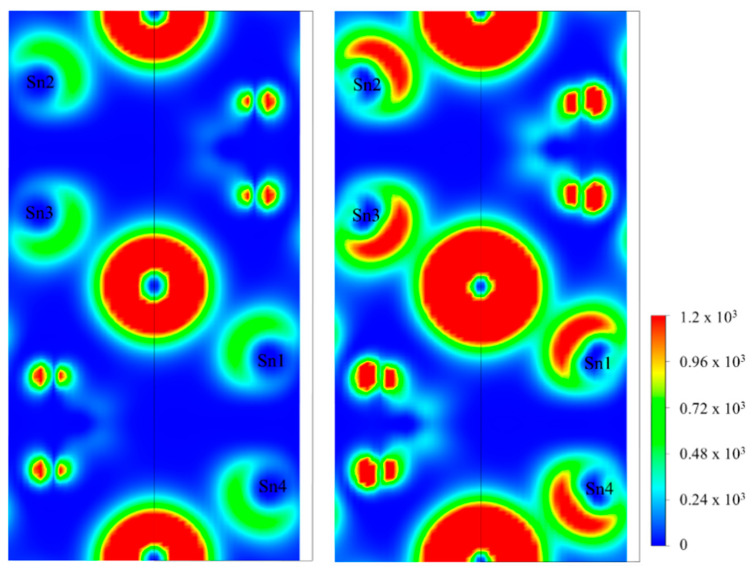
The crystal orbitals in the [−2, 0] eV energy region below the Fermi level of Sn_2_PO_4_I with (**right panel**) and without (**left panel**) the inclusion of spin–orbit effects.

The crystal orbitals near Fermi level [−2, 0] eV were also checked to investigate the stereochemically active lone-pair electrons around Sn(II) cations. As shown in Figure 7, asymmetric lone-pair electrons are also found around the Sn(II) cation. Furthermore, slightly enhanced stereochemically active lone-pair electrons are also found after the inclusion of the spin–orbit effect (shown in Figure 7). The slightly enhanced stereochemically active lone-pair electrons may originate from the splitting of the VB after the inclusion of spin–orbit effects (described above).

Subsequently, the refractive indices and birefringence of Sn_2_PO_4_I were further investigated. As shown in Table 1 and Figure 4, without the inclusion of the spin–orbit effect, the obtained refractive indices of Sn_2_PO_4_I are 2.458 (n_x_), 2.718 (n_y_), and 2.304 (n_z_) @ 1064 nm, and the birefringence is 0.414 @ 1064 nm. After the inclusion of the spin–orbit effect, the refractive indices of Sn_2_PO_4_I are enhanced to 2.644 (n_x_), 2.888 (n_y_), and 2.460 (n_z_) @ 1064 nm, and the birefringence is enhanced to 0.428 @ 1064 nm, which is closer to the results reported in [29] (0.468 @ 1064). It is hypothesized that the enhanced refractive indices and birefringence should have a relation with the reduction in bandgap and stereochemical lone-pair electrons originating from the spin–orbit effect. The astonishing thing is that although the reduction in bandgap is just 0.097 eV, the birefringence is largely enhanced (from 0.414 to 0.428). Therefore, does the reduction in bandgap or the enhanced stereochemical activity of lone pairs make the main contribution to the enhanced birefringence? To answer this question, we further investigated the electronic structures and optical properties of X_2_PO_4_ (X = Ba and Sr), which should not contain stereochemically active lone-pair electrons around Ba(II) or Sr(II) cations.

### 3.3. The Electronic Structures, Refractive Indices, and Birefringence of X_2_PO_4_ (X = Ba, and Sr)

**Figure 8:**
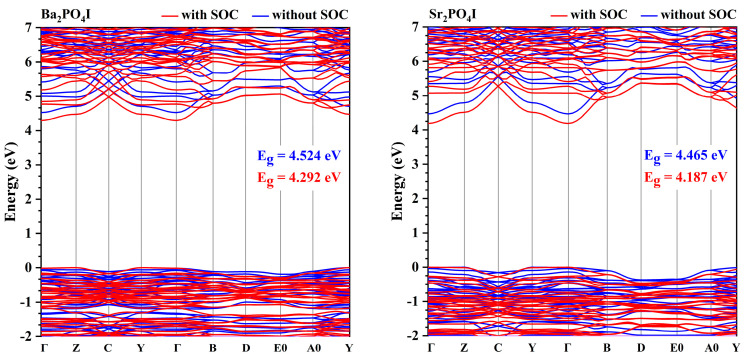
The band structure of Ba_2_PO_4_I and Sr_2_PO_4_I along symmetry directions in the first Brillouin zone with (red color) and without (blue color) the inclusion of spin–orbit effects.

It is noteworthy that the splitting of the bands is also found in X_2_PO_4_ (X = Ba and Sr) belonging to the alkaline earth metals as isomers. Figure 8 and Figure S5 present the obtained band structures of X_2_PO_4_ (X = Ba and Sr) with and without the inclusion of spin–orbit effects, respectively. As shown in Figure 8, without the inclusion of spin–orbit effects, Ba_2_PO_4_I is an indirect bandgap semiconductor with bandgap of 4.524 eV. The top of the VB is located at point Z, and the bottom of the CB is located at point Γ. After the inclusion of spin–orbit effects, Ba_2_PO_4_I is still an indirect bandgap semiconductor with a reduced bandgap of 4.292 eV. Like Ba_2_PO_4_I, Sr_2_PO_4_I is also an indirect bandgap semiconductor with a bandgap of 4.465 eV (without the inclusion of spin–orbit effects) and 4.187 eV (with the inclusion of spin–orbit effects). The top of the VB is located at point Z, and the bottom of the CB is located at point Γ.

**Figure 9:**
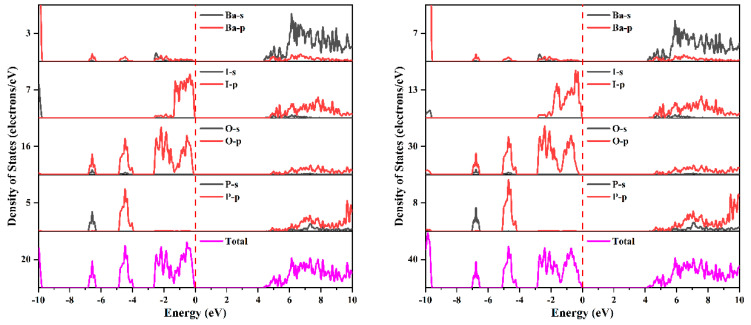
The projected density of states of Ba_2_PO_4_I without (**left panel**) and with (**right panel**) the inclusion of spin–orbit effects.

**Figure 10:**
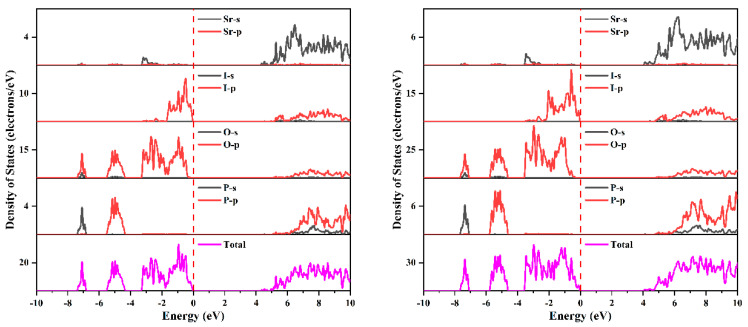
The projected density of states of Sr_2_PO_4_I without (**left panel**) and with (**right panel**) the inclusion of spin–orbit effects.

The splitting of the band should come from the spin–orbit effect of I-p states and cation-p states. Figure 9 and Figure 10 present the projected density of states of Ba_2_PO_4_I and Sr_2_PO_4_I, respectively, and Figures S6 and S7 give the projected band structures of Ba_2_PO_4_I, and Sr_2_PO_4_I, respectively. As shown in Figure 9 and Figure S6, for Ba_2_PO_4_I, there are mainly P-p states, O-p states, and I-p states at the top of the VB, and the main component of the bottom of the CB comes from O-p states, I-p states, and Ba-p states. The splitting of the band near the Fermi level (shown in Figure 9 and Figure S6) may originate from the spin–orbit effect of I-p states, and the splitting of the band at the bottom of CB comes from the spin–orbit effects of Ba-p and I-p states. The combined analysis suggests that the spin–orbit effect in Ba_2_PO_4_I mainly comes from the heavier I elements. Similar conclusions can also be found in Sr_2_PO_4_I (shown in Figure 10 and Figure S7).

What is surprising is that the spin–orbit effect can enhance the refractive indices but attenuate the birefringence of X_2_PO_4_I (X = Ba, and Sr). As shown in Table 1 and Figure 4, for Ba_2_PO_4_I, the refractive indices are 2.059 (n_x_), 2.040 (n_y_), and 2.082 (n_z_) at 1064 nm, which are larger than the results without the inclusion of the SOC, which are 1.895 (n_x_), 1.875 (n_y_), and 1.920 (n_z_). The birefringence with the inclusion of the SOC (about 0.042) is smaller than the result without the inclusion of the SOC (about 0.045). A similar conclusion can also be found in Sr_2_PO_4_I. The enhanced refractive indices and attenuated birefringence found in X_2_PO_4_I (X = Ba and Sr) indicate that without the stereochemically active lone-pair electrons, the change in band structures originating from the spin–orbit effect plays an important role in determining the refractive indices and birefringence.

From the above band analysis, it is clear that the band structure with the inclusion of the SOC effect exhibits the following two changes: band splitting and band reduction. Here, in order to deeply understand the role played by the reduction in bandgap in birefringence, we adopt a method of moving only the conduction bands, the so-called “*shifting of conduction bands*”, which does not renormalize the momentum matrix elements. It is worth noting that this method is distinguished from the scissor operation [47]. This is because the scissor operation, although it can move all the guide bands in order to agree with the measured value of the band gap, causes the momentum matrix elements to be renormalized. Then, upshifting the CB obtained by the inclusion of spin–orbit effects makes it have same bandgap as the one obtained without the inclusion of spin–orbit effects. Using this strategy, except for the bandgap, nothing of the band structures is changed. After this strategy was used, the refractive indices of these compounds with the inclusion of SOC effect were still larger than the values without the inclusion of SOC effect. For example, after this strategy was used, the obtained refractive indices of Pb_2_PO_4_I were 2.453 (n_x_), 2.406 (n_y_), and 2.500 (n_z_), which are larger than the refractive indices without the inclusion of spin–orbit effects, namely 2.393 (n_x_), 2.337 (n_y_), and 2.450 (n_z_) @ 1064 nm (shown in Table 1). Unlike refractive indices, birefringence is attenuated after the ‘*shifting of conduction bands*’. The obtained birefringence values 0.094 (Pb_2_PO_4_I), 0.403 (Sn_2_PO_4_I), 0.037 (Ba_2_PO_4_I), and 0.039 (Sr_2_PO_4_I), which are smaller than the results without the inclusion of SOC effects, namely 0.113 (Pb_2_PO_4_I), 0.414 (Sn_2_PO_4_I), 0.045 (Ba_2_PO_4_I), and 0.049 (Sr_2_PO_4_I).

In short, according to the results described above, one can deduce that the spin–orbit effects of 5p and 6p states could lead to the splitting of the band, reductions in bandgap, enhancement of stereochemical activity of lone pairs, and changes in refractive indices and birefringence. The change in band structure plays a more important role in determining the enhancement of birefringence than the slightly enhanced stereochemical activity of lone pairs.

## 4. Conclusions

In conclusion, the electronic structure, refractive indices, and birefringence of a series of X_2_PO_4_I (X = Pb, Sn, Ba, and Sr) compounds involving SOC were systematically investigated for the first time using a first-principles approach. Considerable splitting of valence and conduction bands due to spin–orbit effects in the 5p and 6p states was found in these compounds. Analysis showed that the splitting of the bands may arise from the interaction of the spin with heavy element-p orbitals and I-p states. The refractive index and birefringence also appeared to have been altered. It was confirmed that Pb_2_PO_4_I and Sn_2_PO_4_I with lone-pair electron activity showed a slight enhancement of stereochemical activity after considering SOC. Interestingly, the electronic structure, refractive indices, and birefringence of the alkaline earth metal phosphates Ba_2_PO_4_I and Sr_2_PO_4_I, which are isomers of the two compounds mentioned above, confirm that changes in the band structure due to spin–orbit effects play an important role in the refractive index and birefringence. To further analyze this interesting phenomenon, it was found, using the ‘*shifting of conduction bands*’ method, that the effect of changes in the band structure on birefringence enhancement is greater than the slight enhancement of stereochemical activity. The above interesting findings of the systematic analysis of the electronic structure, refractive indices, and birefringence after considering the SOC effect demonstrate the importance of the SOC effect in the study of post-transition metals and provide a new direction for related research.

## Figures and Tables

**Table 1:** The obtained refractive indices and birefringence (@ 1064 nm) of X_2_PO_4_I (X = Pb, Sn, Ba, and Sr) without and with the inclusion of spin–orbit effects.

Compound		n_x_	n_y_	n_z_	Δn
Pb_2_PO_4_I	Without SOC	2.393	2.337	2.450	0.113
	With SOC	2.710	2.643	2.774	0.131
Sn_2_PO_4_I	Without SOC	2.458	2.718	2.304	0.414
	With SOC	2.644	2.888	2.460	0.428
Ba_2_PO_4_I	Without SOC	1.895	1.875	1.920	0.045
	With SOC	2.059	2.040	2.082	0.042
Sr_2_PO_4_I	Without SOC	1.876	1.855	1.904	0.049
	With SOC	2.057	2.037	2.083	0.046

## Data Availability

Data are contained within the article.

## Supplementary Materials

The following supporting information can be downloaded at: https://www.mdpi.com/article/10.3390/nano14070617/s1, Figure S1. The band structure of Pb_2_PO_4_I without spin-orbit effects and with spin-orbit effects, within the energy range of −20 eV to 10 eV; Figure S2. The projected band structure of Pb_2_PO_4_I along symmetry directions in the first Brillouin zone without (left panel) and with (right panel) spin-orbit effects; Figure S3. The band structure of Sn_2_PO_4_I without spin-orbit effects and with spin-orbit effects, within the energy range of −20 eV to 10 eV; Figure S4. The projected band structure of Sn_2_PO_4_I along symmetry directions in the first Brillouin zone without (left panel) and with (right panel) spin-orbit effects; Figure S5. The band structure of Ba_2_PO_4_I and Sr_2_PO_4_I without spin-orbit effects and with spin-orbit effects, within the energy range of −20 eV to 10 eV; Figure S6. The projected band structure of Ba_2_PO_4_I along symmetry directions in the first Brillouin zone without (left panel) and with (right panel) spin-orbit effects; Figure S7. The projected band structure of Sr_2_PO_4_I along symmetry directions in the first Brillouin zone without (left panel) and with (right panel) spin-orbit effects.
