# Supplementary material for: The Spin–Orbit Effect on the Electronic Structures, Refractive Indices, and Birefringence of X2PO4I (X = Pb, Sn, Ba and Sr): A First-Principles Investigation
Source: Nanomaterials (Basel). 2024 Apr 1;14(7):617. doi: 10.3390/nano14070617 (PMC11013657; doi:10.3390/nano14070617)
Supplement: Supplementary file 1 [file nanomaterials-14-00617-s001.zip › nanomaterials-2926978-supplementary.pdf]

Supplementary Materials

The Spin–Orbit Effect on the Electronic Structures, Refractive Indices, and Birefringence of X_2PO_4I (X = Pb, Sn, Ba and Sr): A First-Principles Investigation

Xudong Leng †, Mei Hu †, Qun Jing *, Haiming Duan, Henglei Chen and Xiuhua Cui *

Xinjiang Key Laboratory of Solid State Physics and Devices, School of Physical Science and Technology,
Xinjiang University, Urumqi 830017, China; chillsir@stu.xju.edu.cn (X.L.); humei09292022@163.com (M.H.); dhm@xju.edu.cn (H.D.); chl@xju.edu.cn (H.C.)
* Correspondence: qunjing@xju.edu.cn (Q.J.); xjcxh0991@xju.edu.cn (X.C.)
† These authors contributed equally to this work.

Figure S1. The band structure of Pb_2PO_4I without spin-orbit effects and with spin-orbit effects, within the energy range of -20 eV to 10 eV.

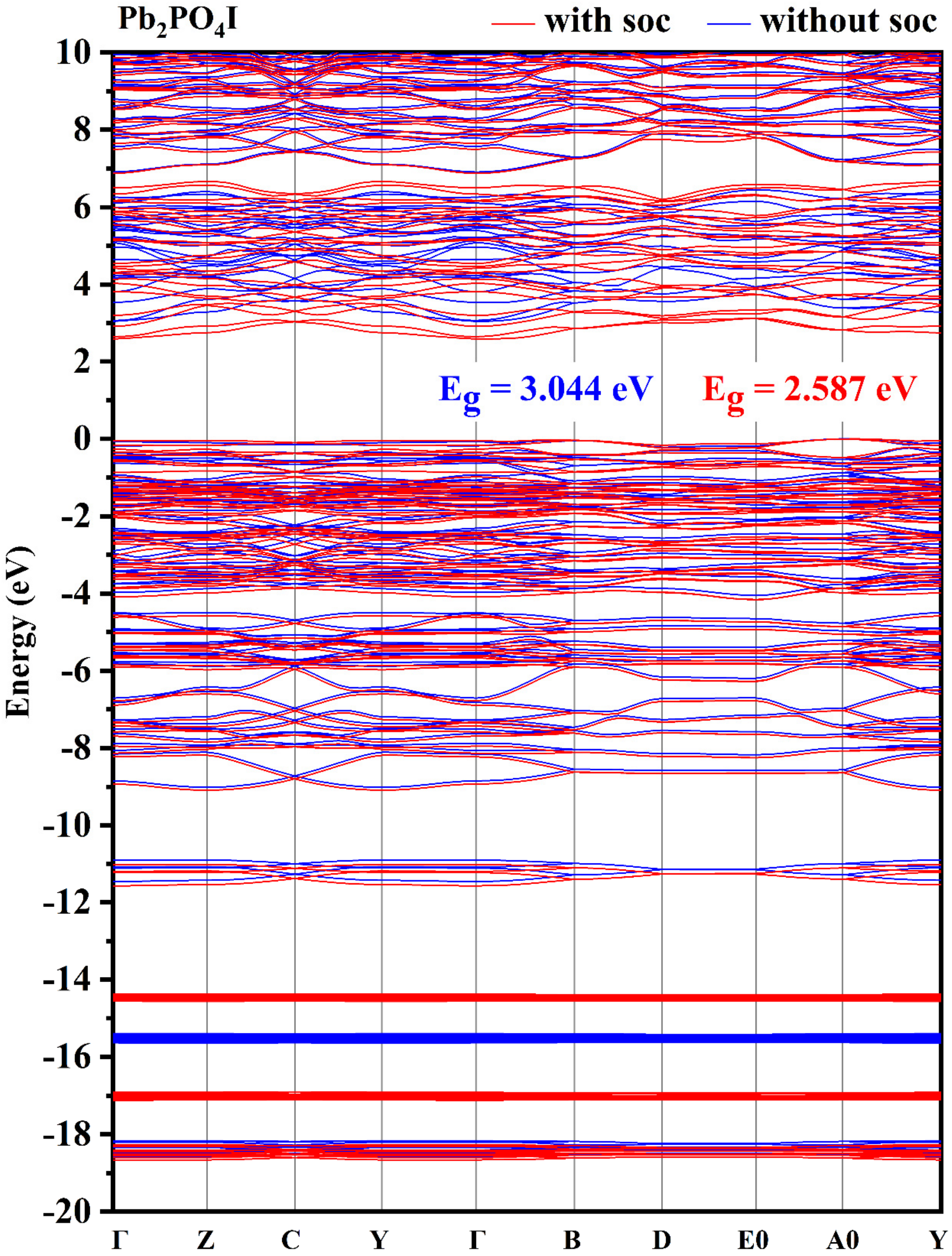

Figure S2. The projected band structure of Pb_2PO_4I along symmetry directions in the first Brillouin zone without (left panel) and with (right panel) spin-orbit effects.

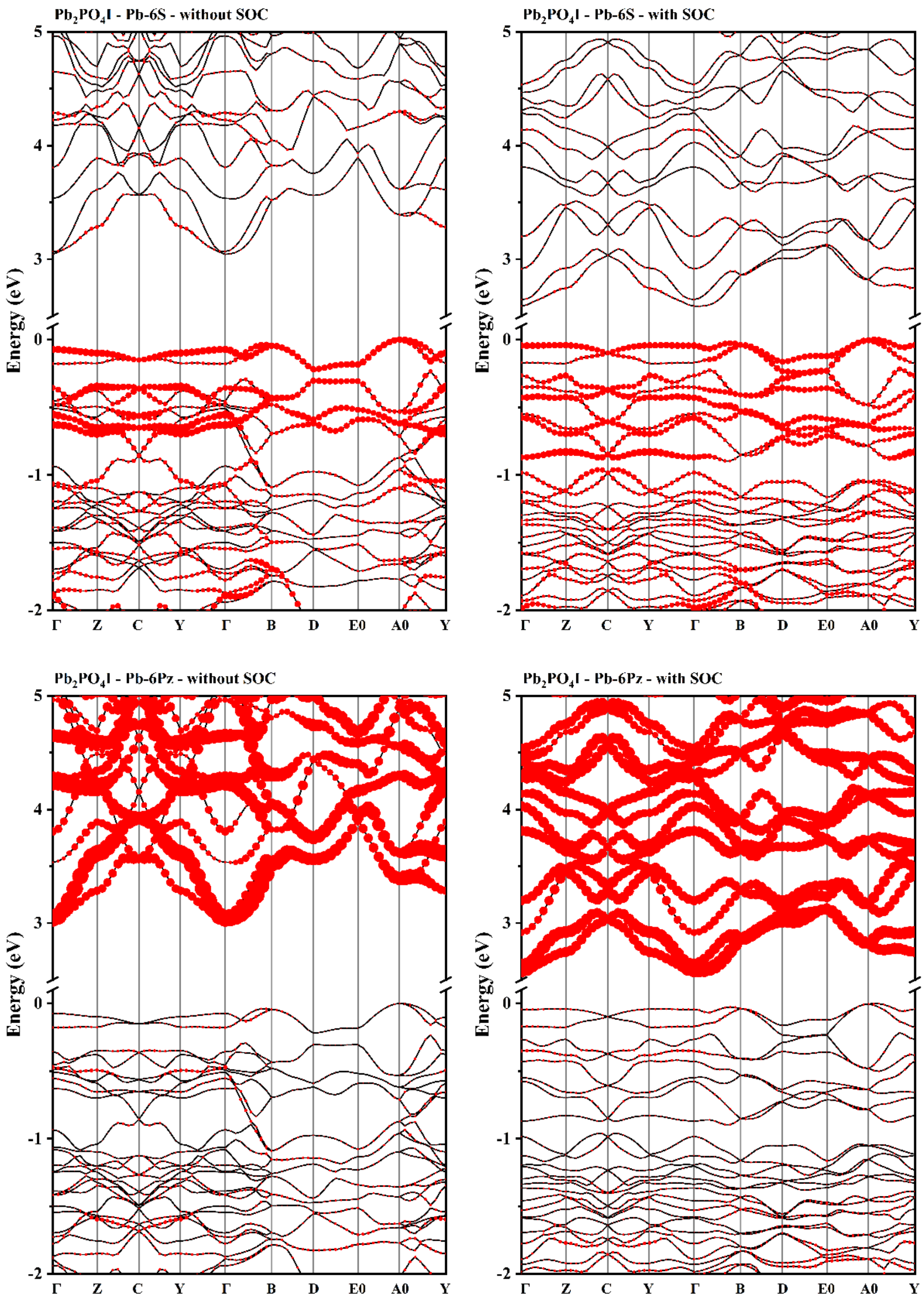

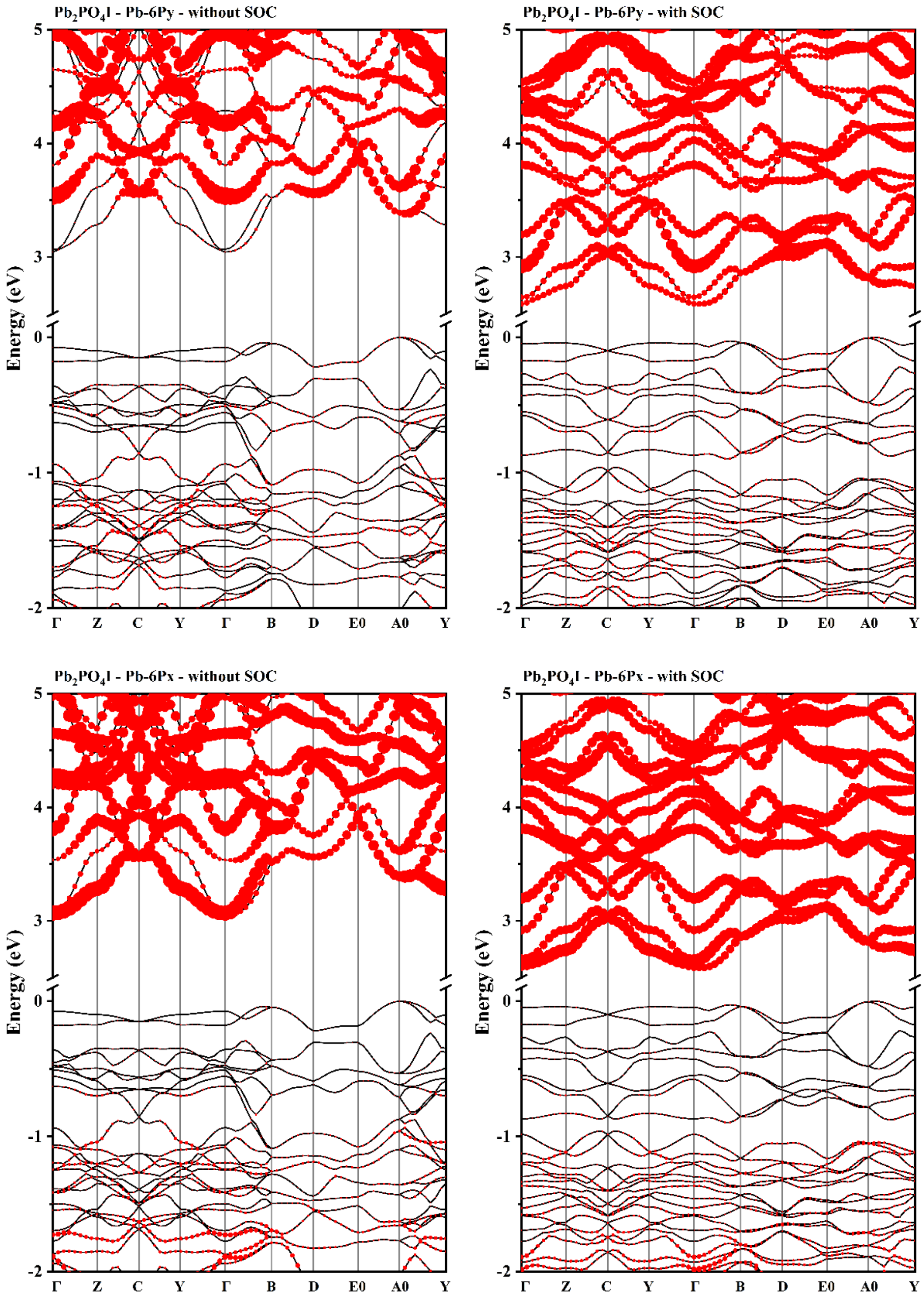
Pb2PO4I - Pb-6Py - without SOC
Pb2PO4I - Pb-6Py - with SOC
Pb2PO4I - Pb-6Px - without SOC
Pb2PO4I - Pb-6Px - with SOC
Energy (eV)
5
4
3
0
-1
-2
Γ Z C Y Γ B D E0 A0 Y

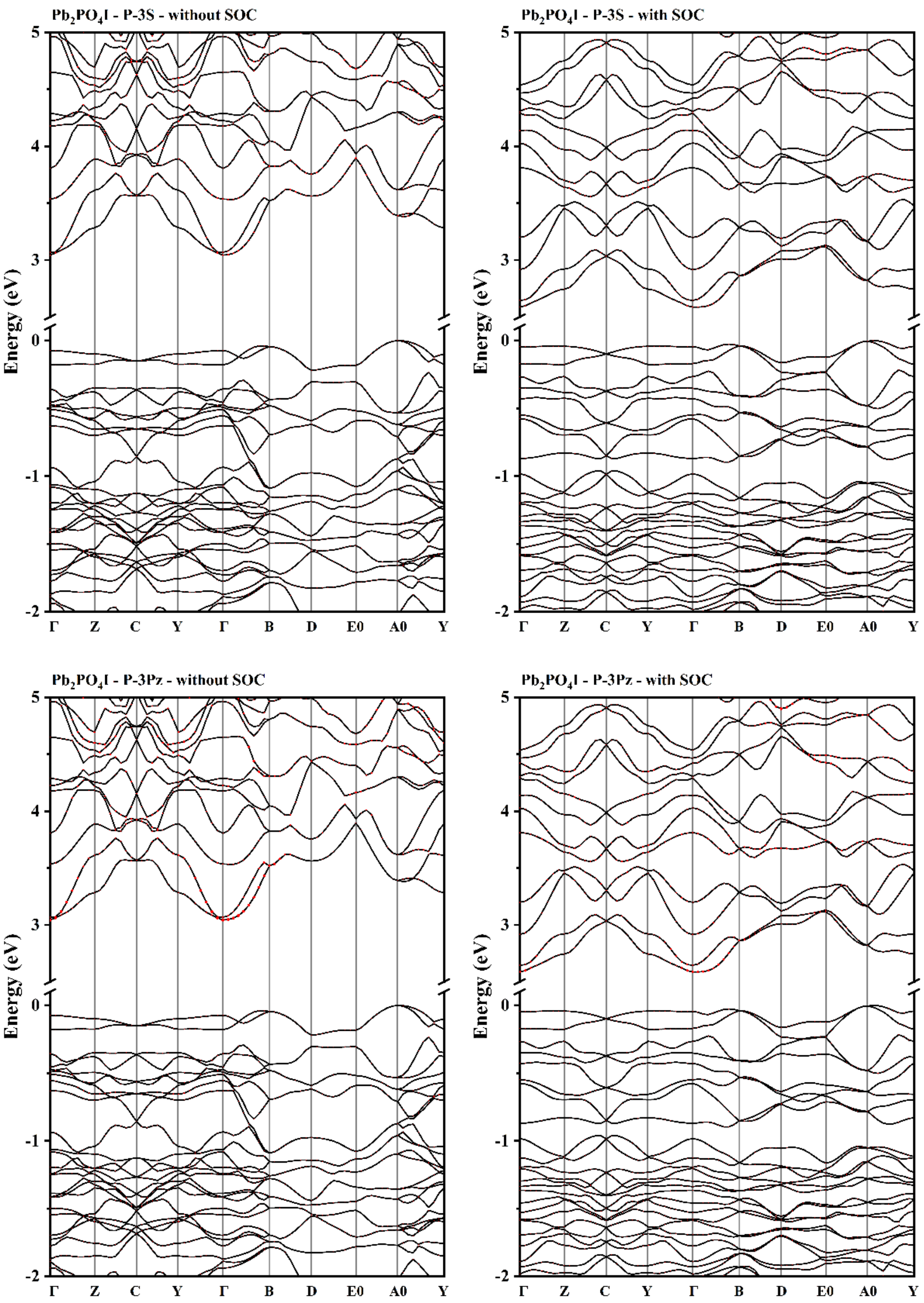

Pb2PO4I - P-3S - without SOC
Pb2PO4I - P-3S - with SOC
Pb2PO4I - P-3Pz - without SOC
Pb2PO4I - P-3Pz - with SOC
Energy (eV)
5
4
3
0
-1
-2
Γ Z C Y Γ B D E0 A0 Y

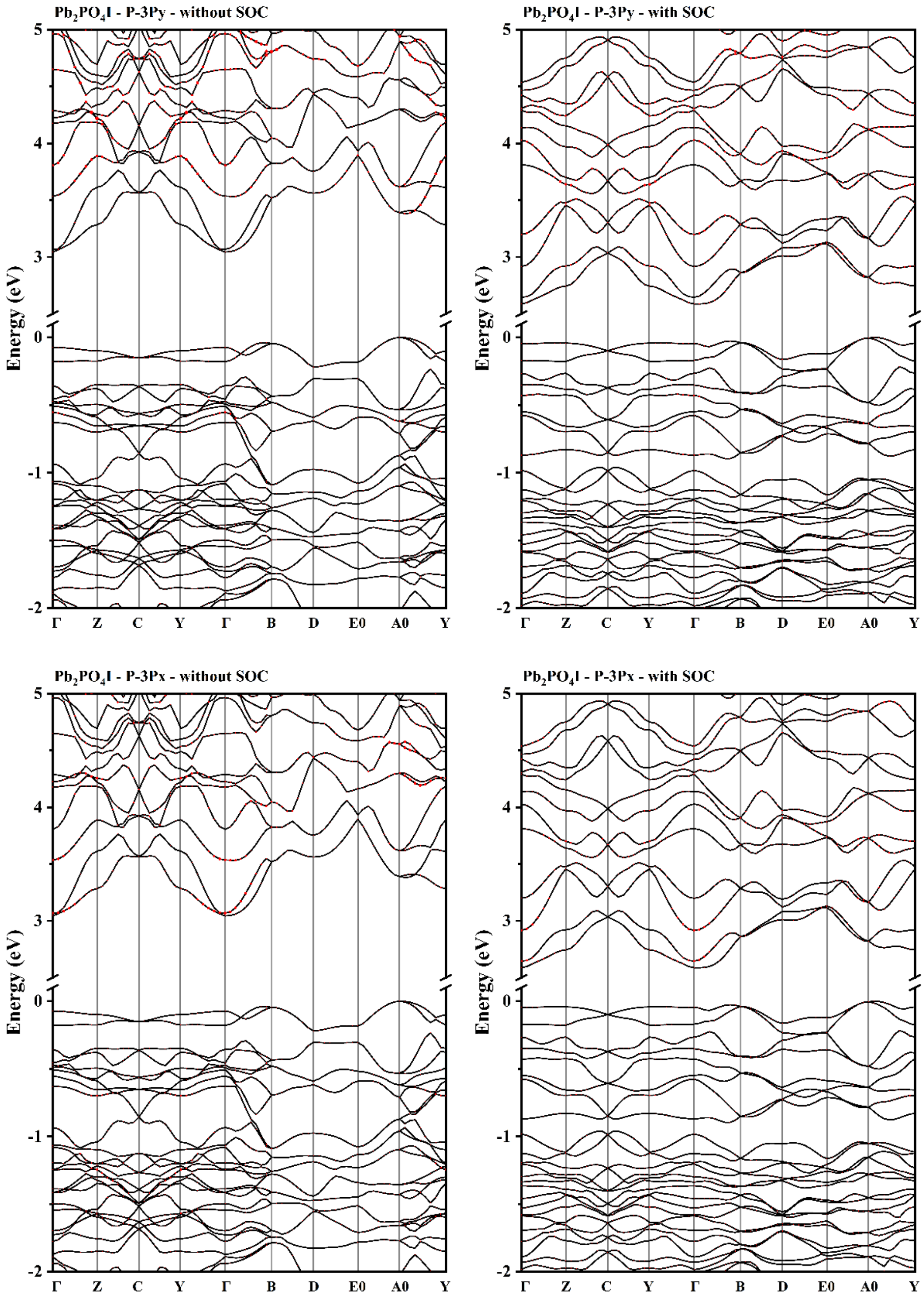
Pb2PO4I - P-3Py - without SOC
Pb2PO4I - P-3Py - with SOC
Pb2PO4I - P-3Px - without SOC
Pb2PO4I - P-3Px - with SOC
Energy (eV)
5
4
3
0
-1
-2
Γ Z C Y Γ B D E0 A0 Y

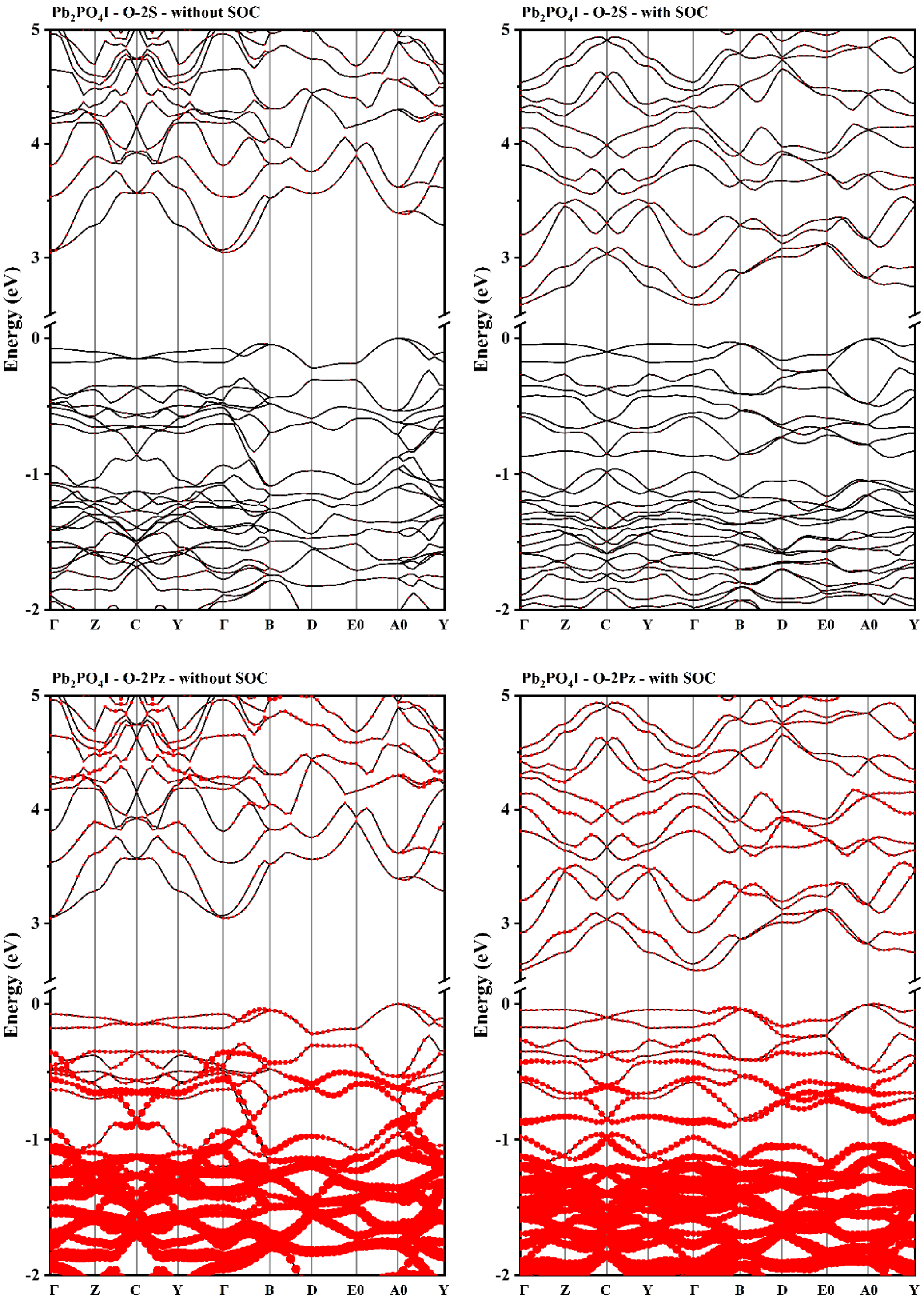

Pb$_2$PO$_4$I - O-2S - without SOC
Pb$_2$PO$_4$I - O-2S - with SOC
Pb$_2$PO$_4$I - O-2Pz - without SOC
Pb$_2$PO$_4$I - O-2Pz - with SOC
Energy (eV)
5
4
3
0
-1
-2
Γ Z C Y Γ B D E0 A0 Y

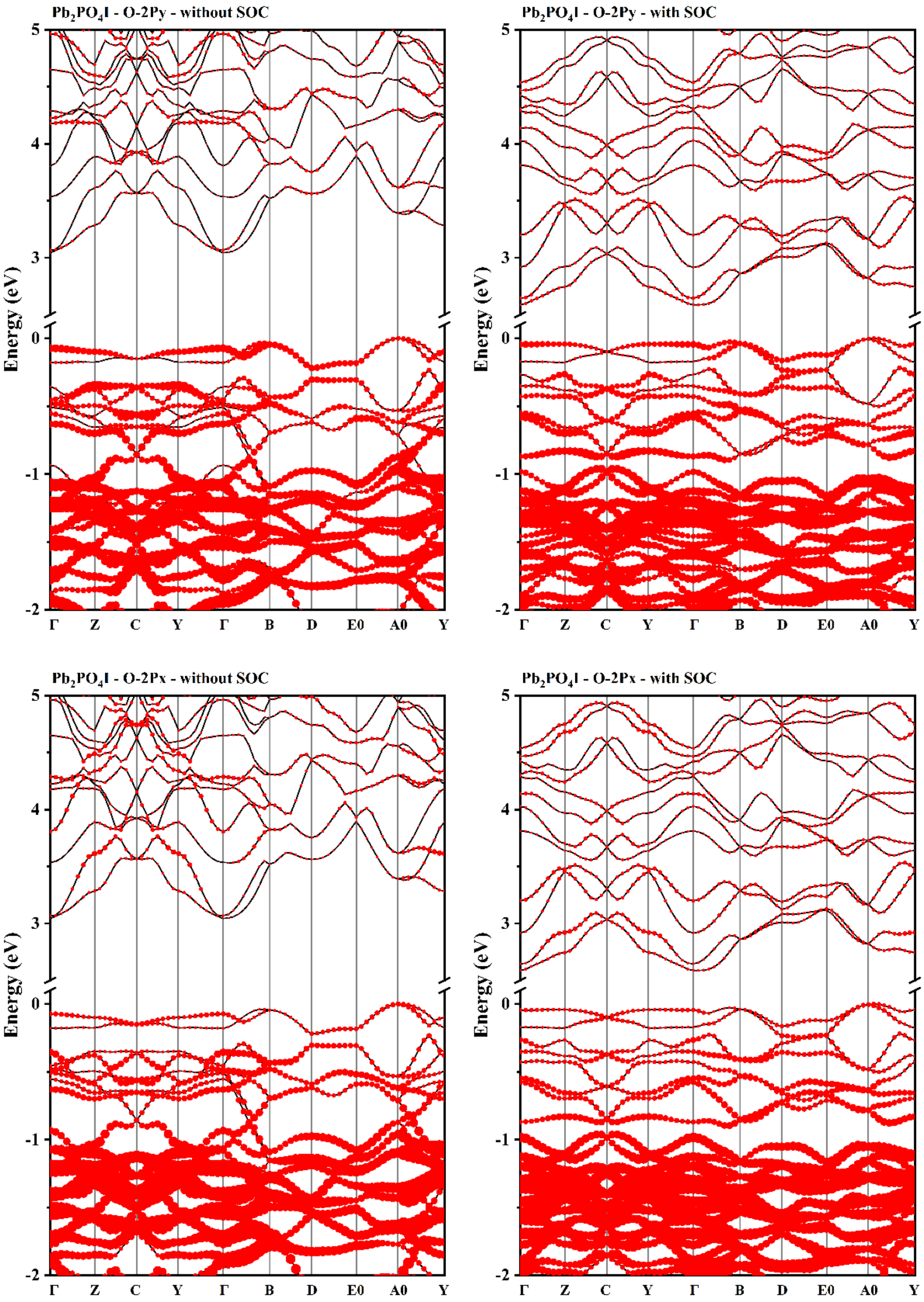

Pb2PO4I - O-2Py - without SOC
Pb2PO4I - O-2Py - with SOC
Pb2PO4I - O-2Px - without SOC
Pb2PO4I - O-2Px - with SOC
Energy (eV)
5
4
3
0
-1
-2
Γ Z C Y Γ B D E0 A0 Y

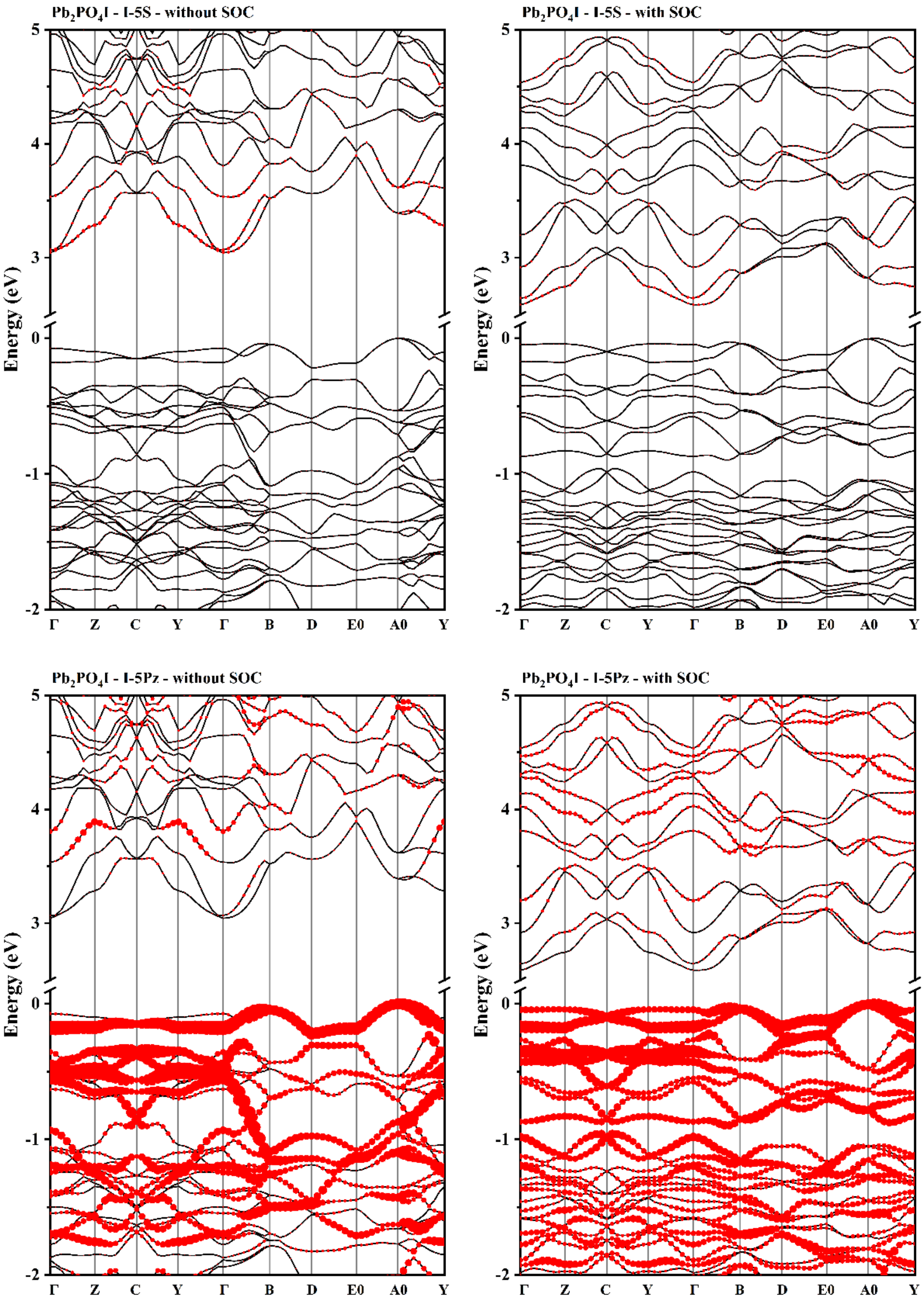

Pb2PO4I - I-5S - without SOC
Pb2PO4I - I-5S - with SOC
Pb2PO4I - I-5Pz - without SOC
Pb2PO4I - I-5Pz - with SOC
Energy (eV)
5
4
3
0
-1
-2
Γ Z C Y Γ B D E0 A0 Y

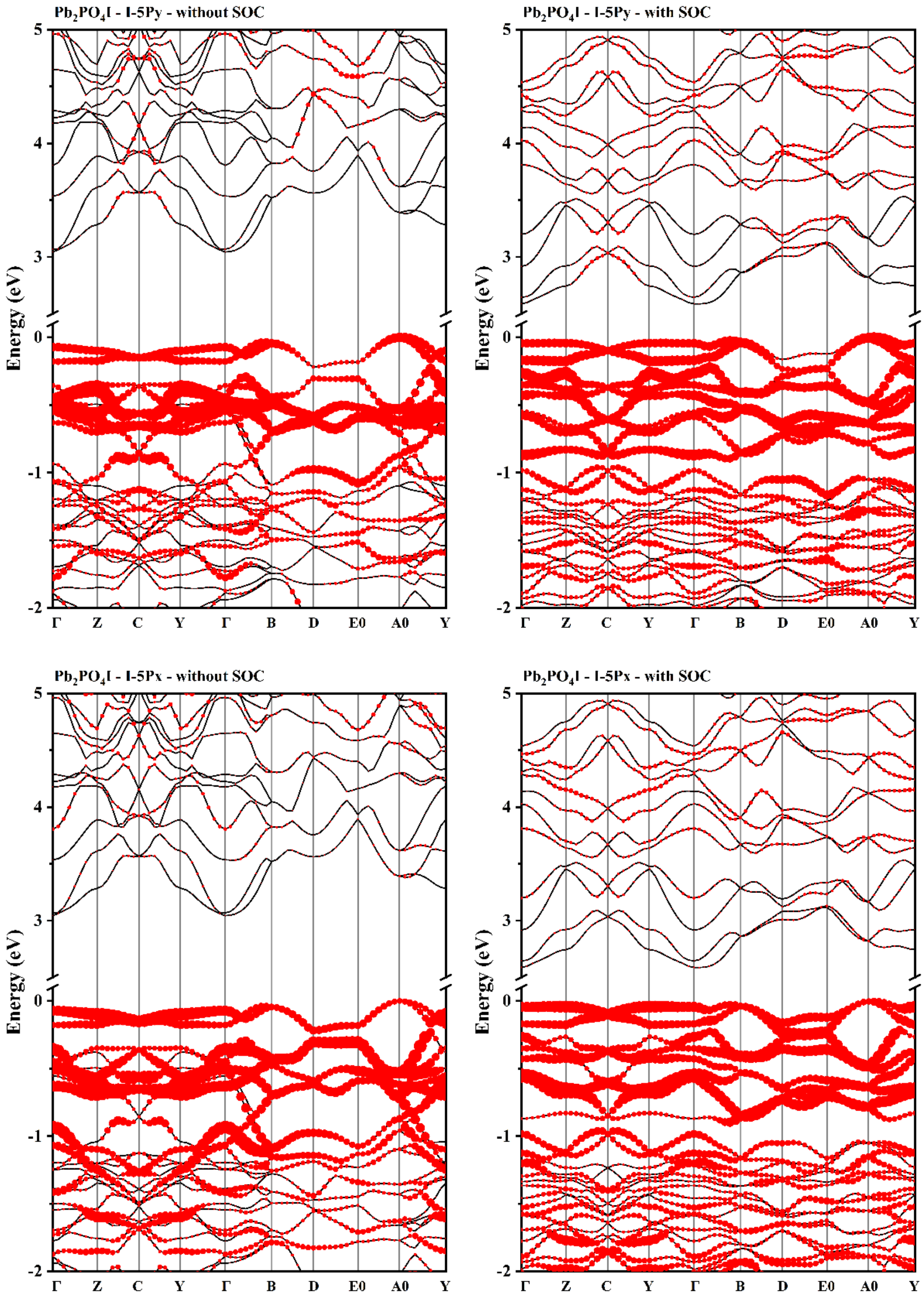

Pb2PO4I - I-5Py - without SOC
Pb2PO4I - I-5Py - with SOC
Pb2PO4I - I-5Px - without SOC
Pb2PO4I - I-5Px - with SOC
Energy (eV)
5
4
3
0
-1
-2
Γ
Z
C
Y
Γ
B
D
E0
A0
Y

Figure S3. The band structure of Sn_2PO_4I without spin-orbit effects and with spin-orbit effects, within the energy range of -20 eV to 10 eV.

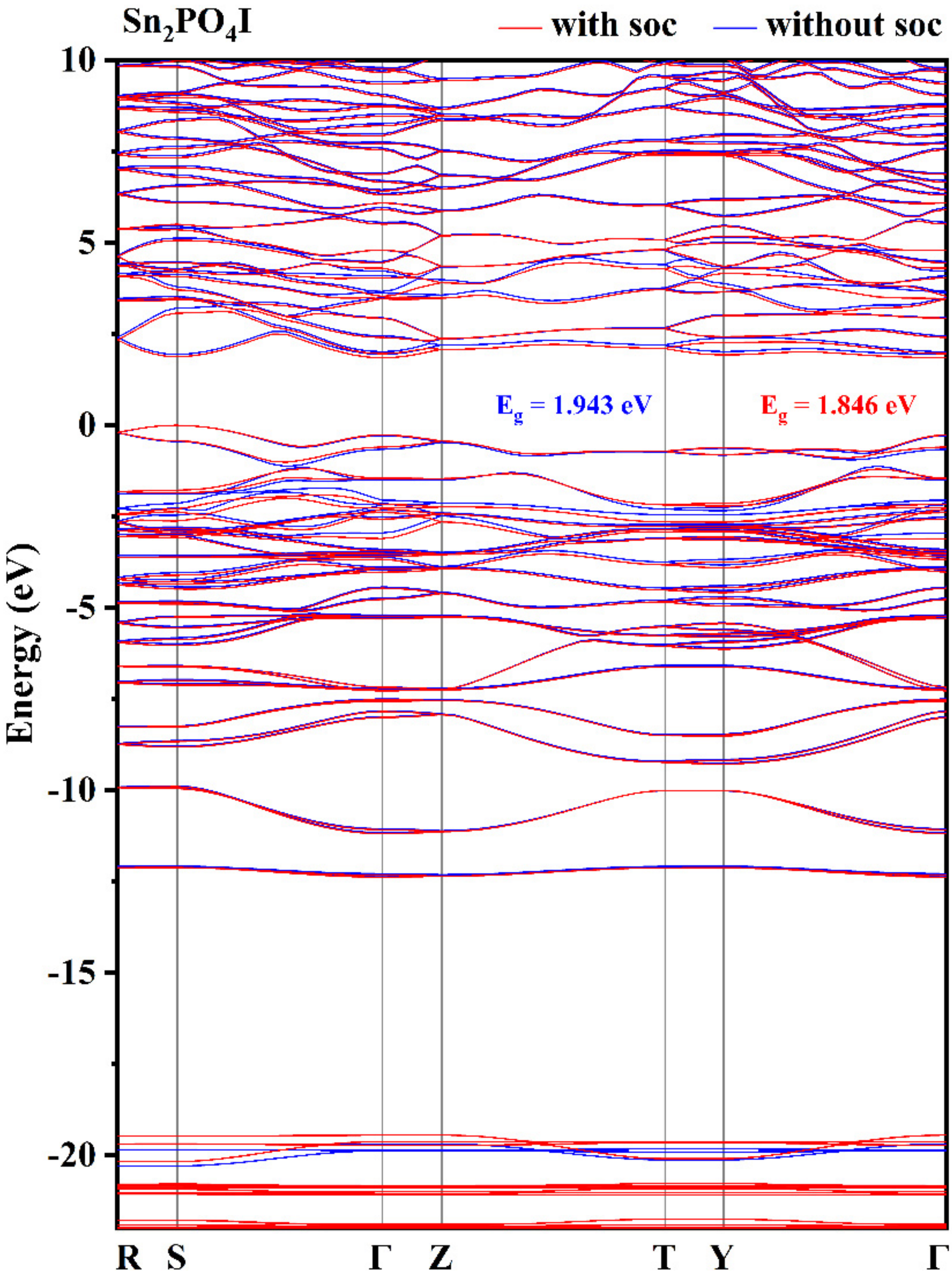

Figure S4. The projected band structure of Sn_2PO_4I along symmetry directions in the first Brillouin zone without (left panel) and with (right panel) spin-orbit effects.

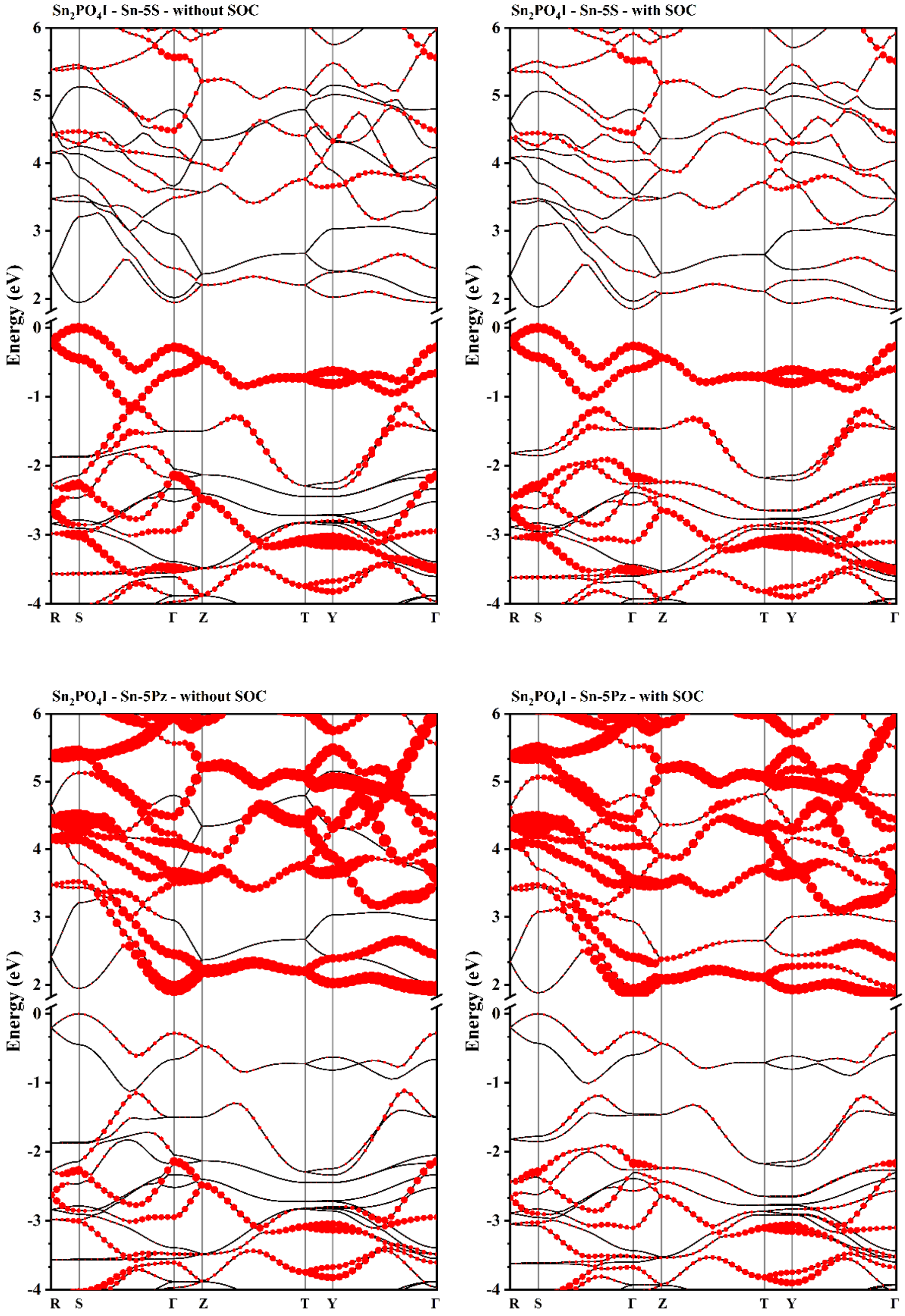

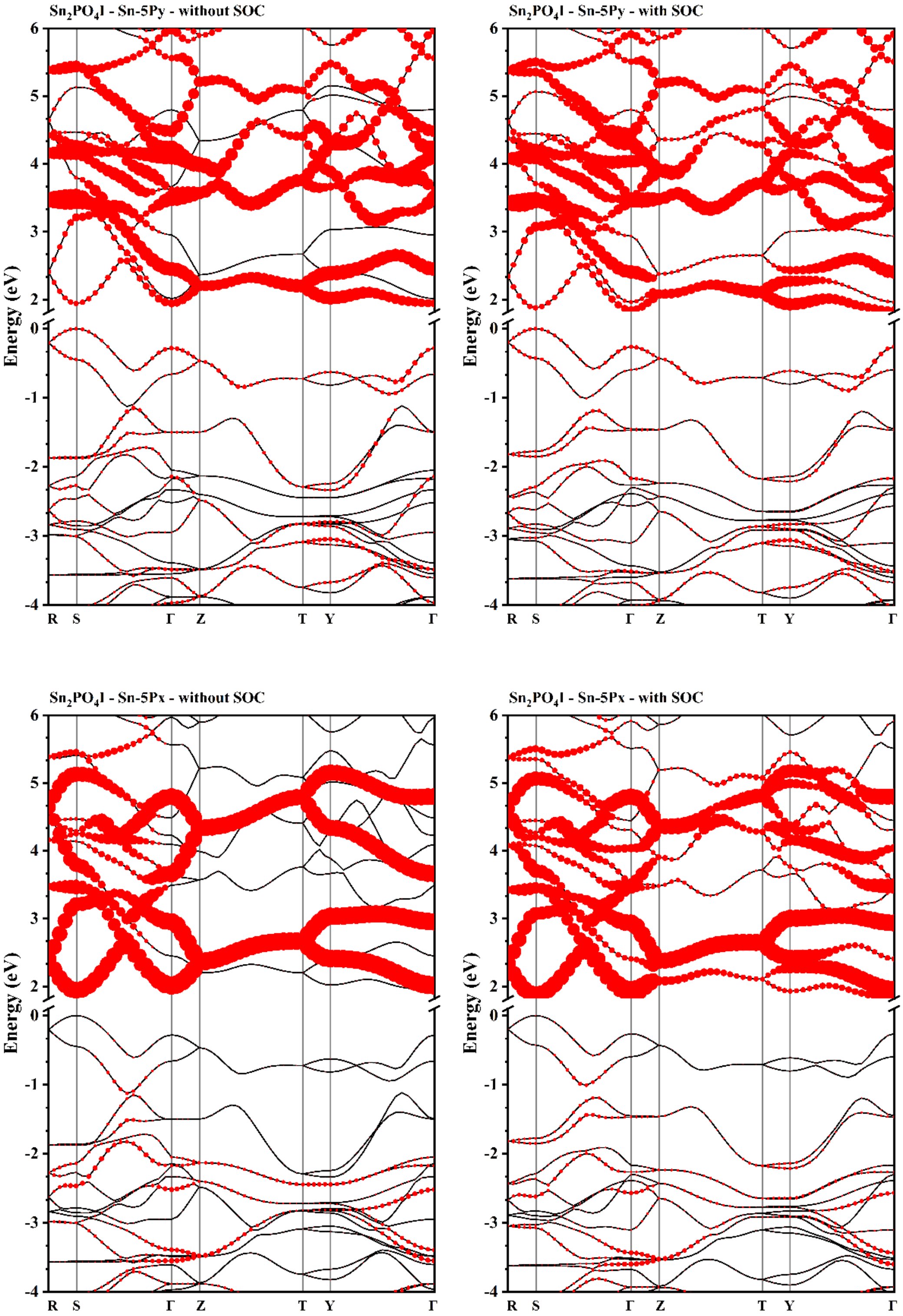

Sn_2PO_4I - Sn-5Py - without SOC
Sn_2PO_4I - Sn-5Py - with SOC
Sn_2PO_4I - Sn-5Px - without SOC
Sn_2PO_4I - Sn-5Px - with SOC
Energy (eV)
6
5
4
3
2
0
-1
-2
-3
-4
R S Γ Z T Y Γ

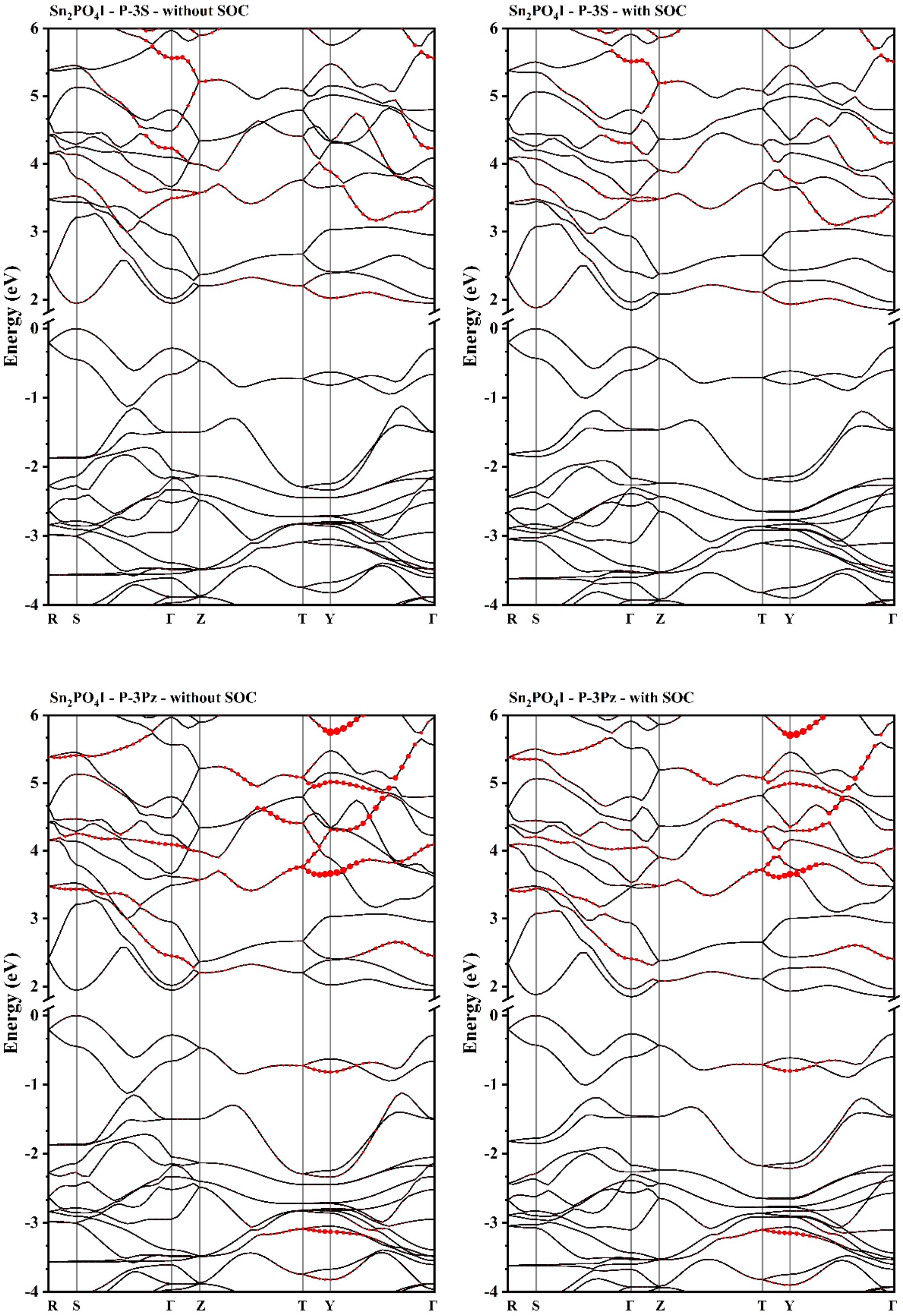

Sn2PO4I - P-3S - without SOC
Sn2PO4I - P-3S - with SOC
Sn2PO4I - P-3Pz - without SOC
Sn2PO4I - P-3Pz - with SOC
Energy (eV)
6
5
4
3
2
0
-1
-2
-3
-4
R S Γ Z T Y Γ

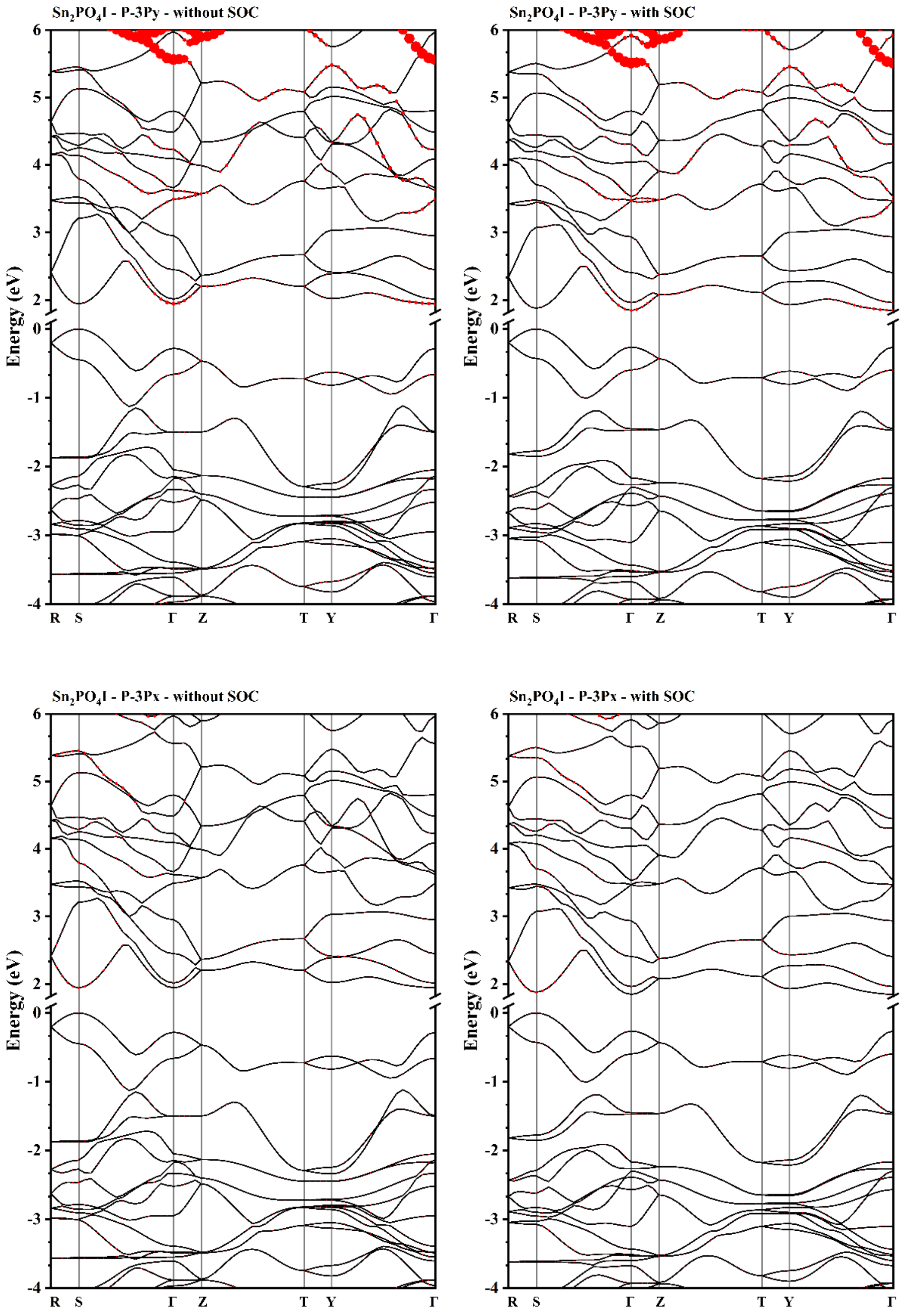
Sn2PO4I - P-3Py - without SOC
Sn2PO4I - P-3Py - with SOC
Sn2PO4I - P-3Px - without SOC
Sn2PO4I - P-3Px - with SOC
Energy (eV)
6
5
4
3
2
0
-1
-2
-3
-4
R S Γ Z T Y Γ

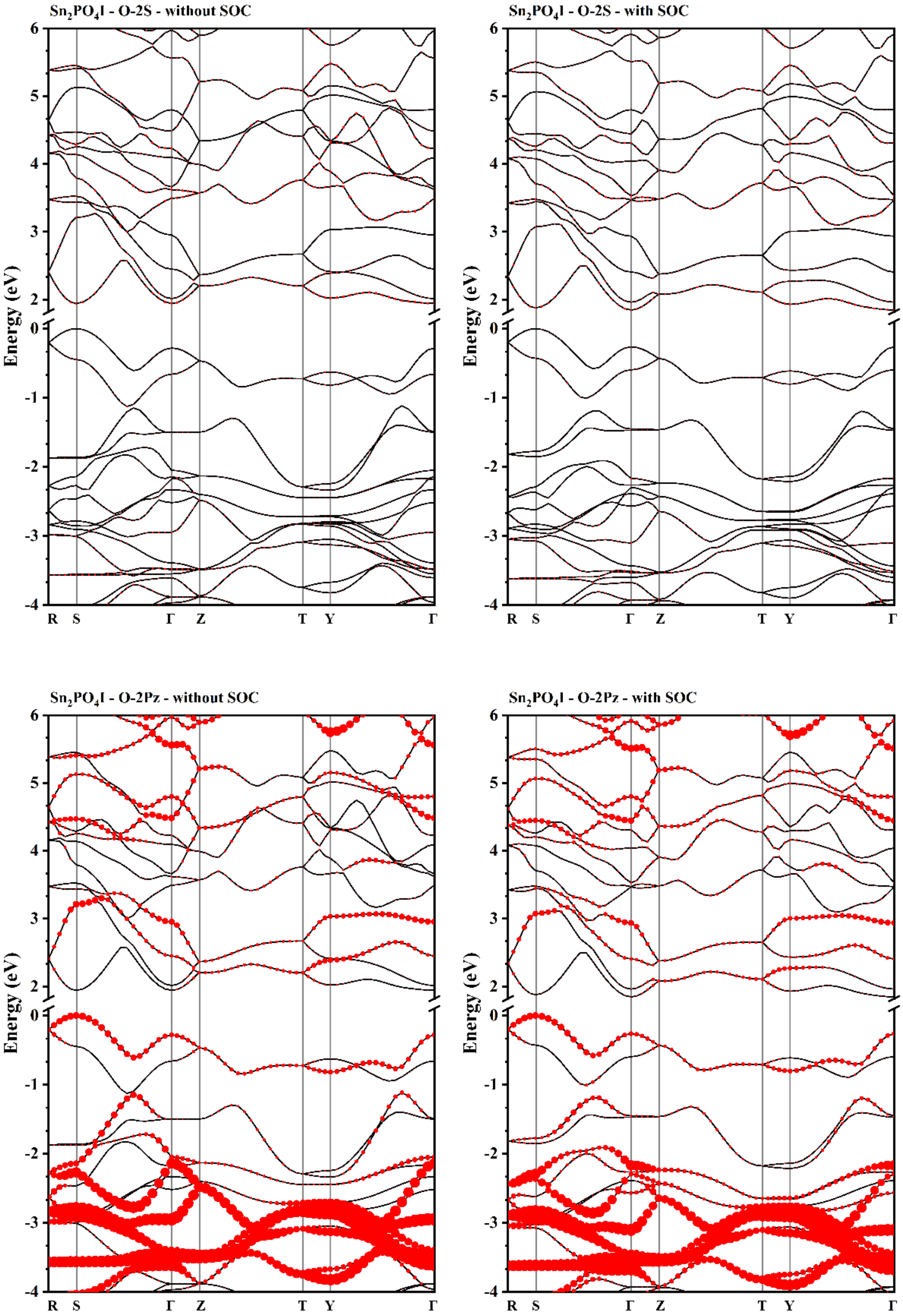
Sn2PO4I - O-2S - without SOC
Sn2PO4I - O-2S - with SOC
Sn2PO4I - O-2Pz - without SOC
Sn2PO4I - O-2Pz - with SOC
Energy (eV)
R S Γ Z T Y Γ
6
5
4
3
2
0
-1
-2
-3
-4

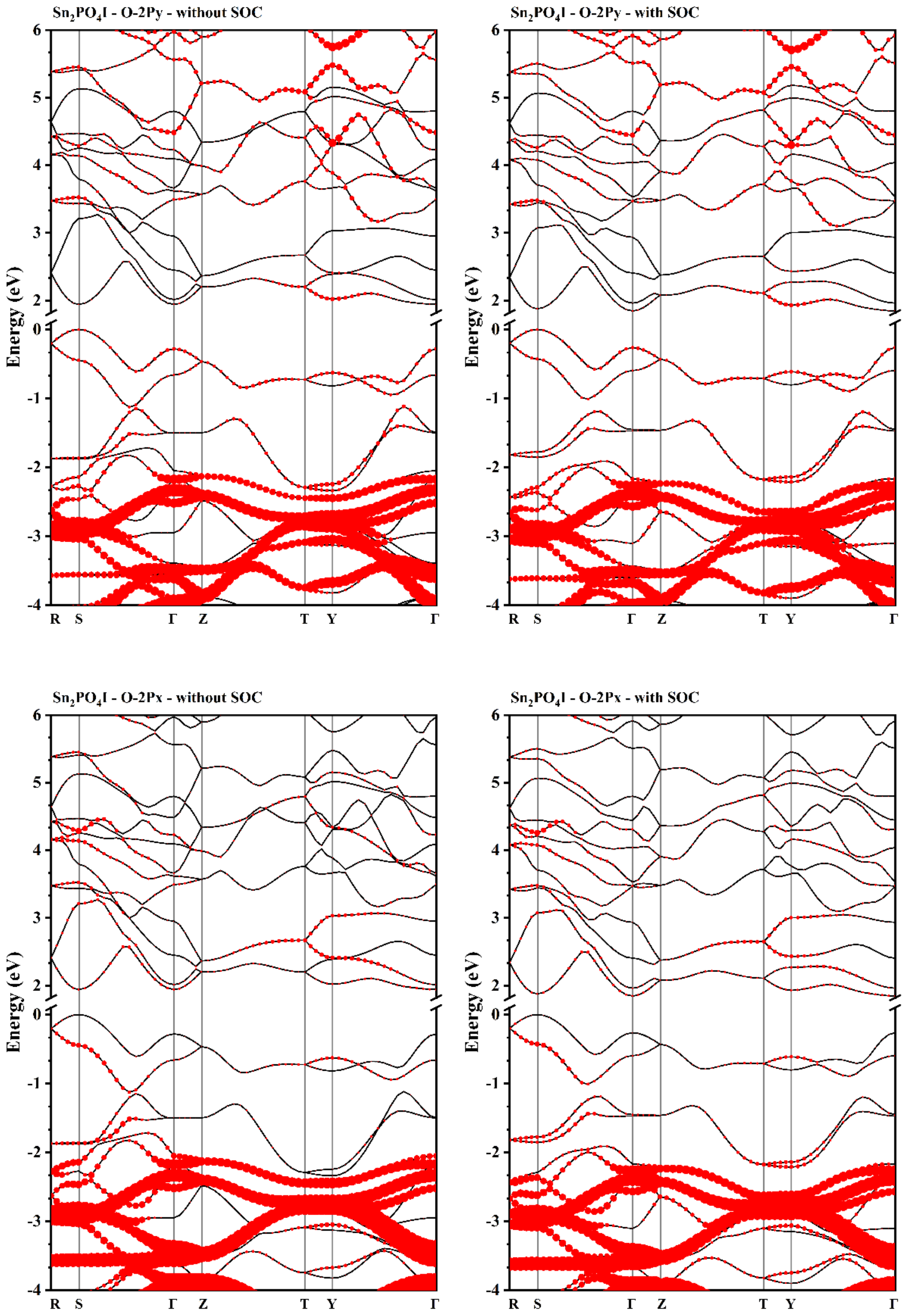

Sn2PO4I - O-2Py - without SOC
Sn2PO4I - O-2Py - with SOC
Sn2PO4I - O-2Px - without SOC
Sn2PO4I - O-2Px - with SOC
Energy (eV)
6
5
4
3
2
0
-1
-2
-3
-4
R S Γ Z T Y Γ

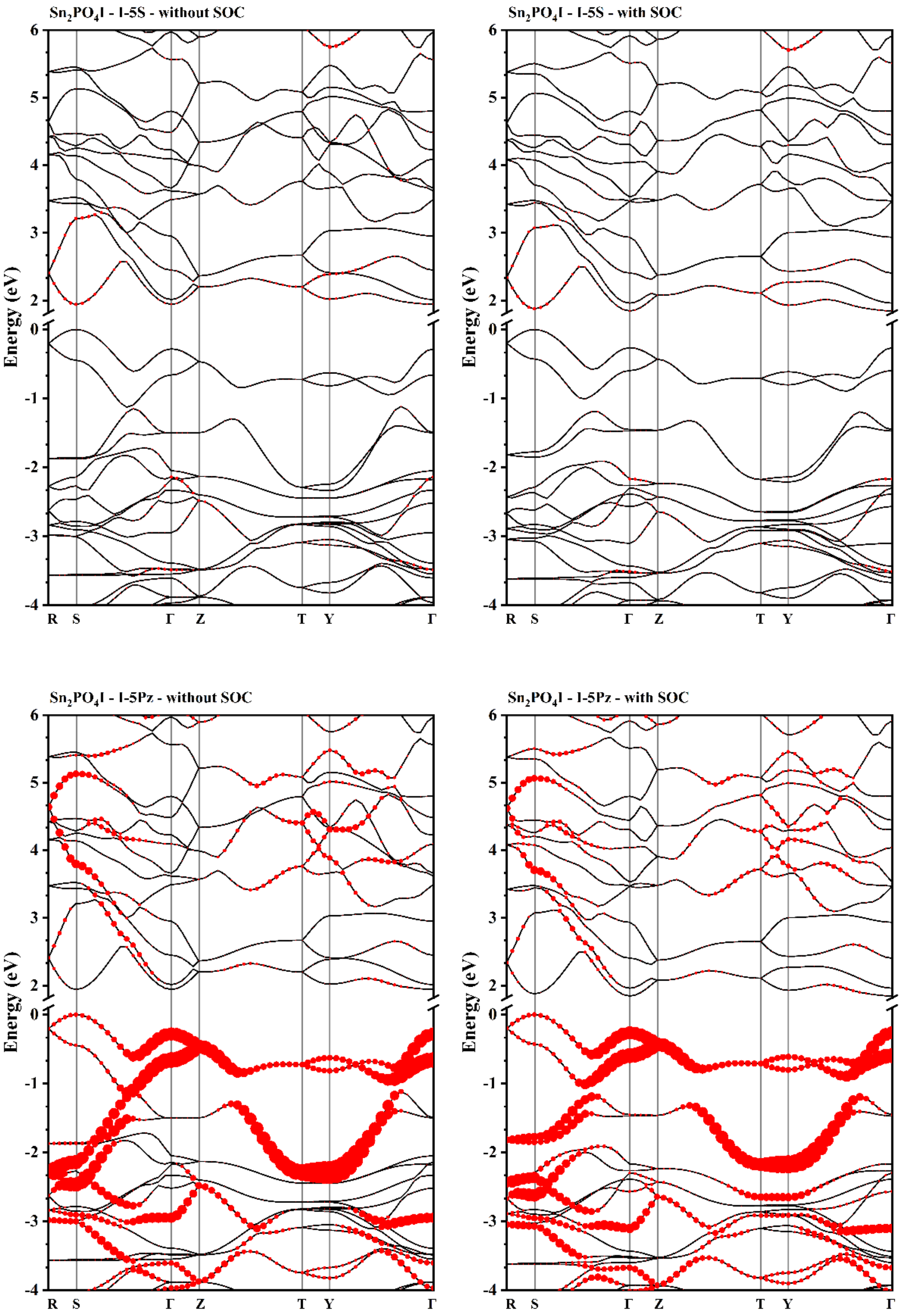

Sn2PO4I - I-5S - without SOC
Sn2PO4I - I-5S - with SOC
Sn2PO4I - I-5Pz - without SOC
Sn2PO4I - I-5Pz - with SOC
Energy (eV)
6
5
4
3
2
0
-1
-2
-3
-4
R
S
Γ
Z
T
Y
Γ

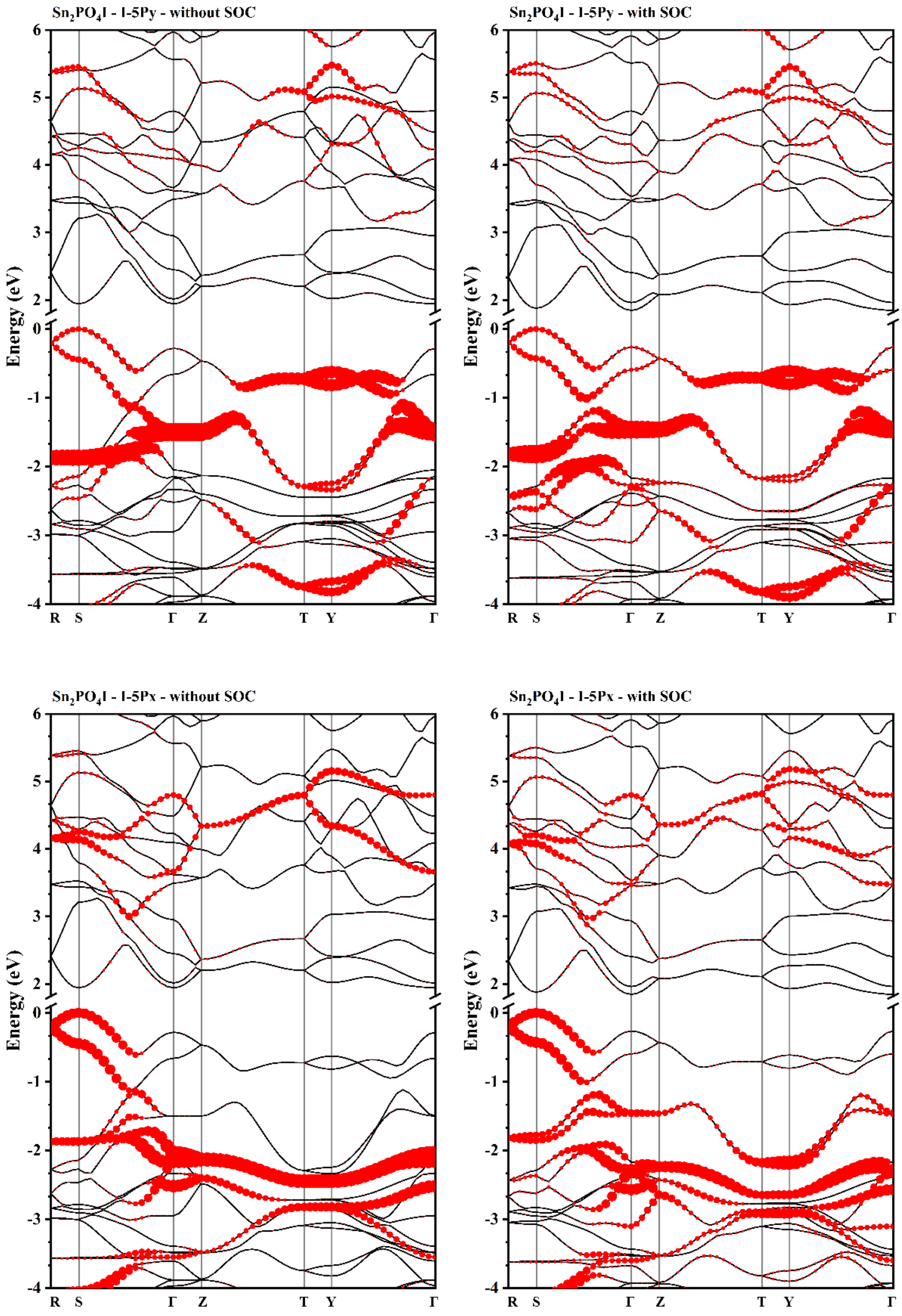

Sn2PO4I - I-5Py - without SOC
Sn2PO4I - I-5Py - with SOC
Sn2PO4I - I-5Px - without SOC
Sn2PO4I - I-5Px - with SOC
Energy (eV)
6
5
4
3
2
0
-1
-2
-3
-4
R S Γ Z T Y Γ

Figure S5. The band structure of Ba_2PO_4I and Sr_2PO_4I without spin-orbit effects and with spin-orbit effects, within the energy range of -20 eV to 10 eV.

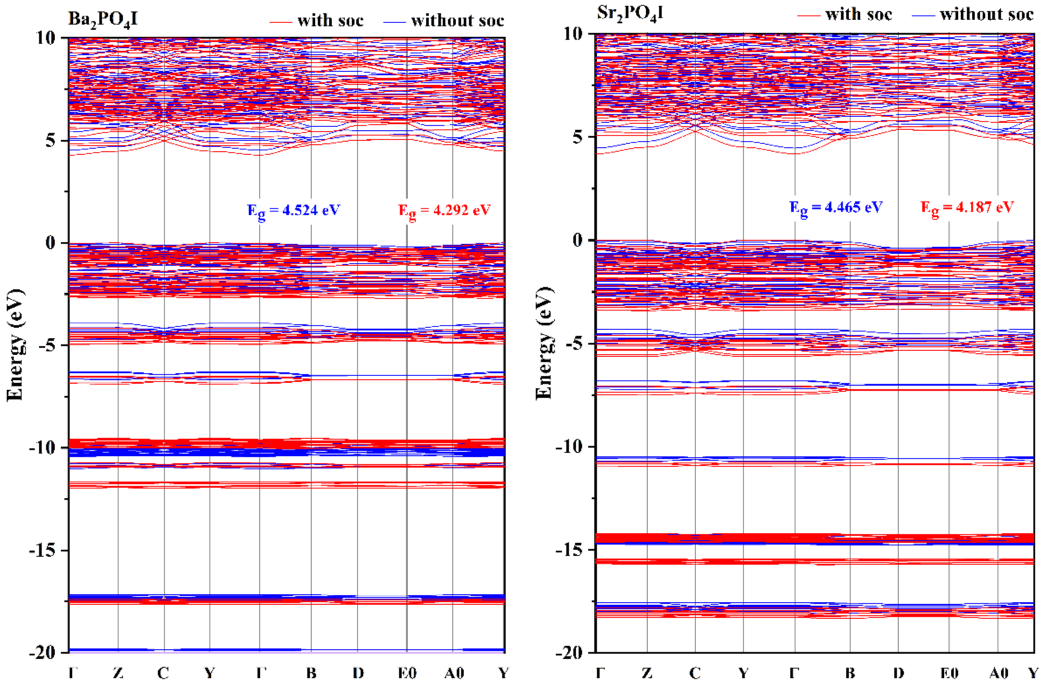

Figure S6. The projected band structure of Ba_2PO_4I along symmetry directions in the first Brillouin zone without (left panel) and with (right panel) spin-orbit effects.

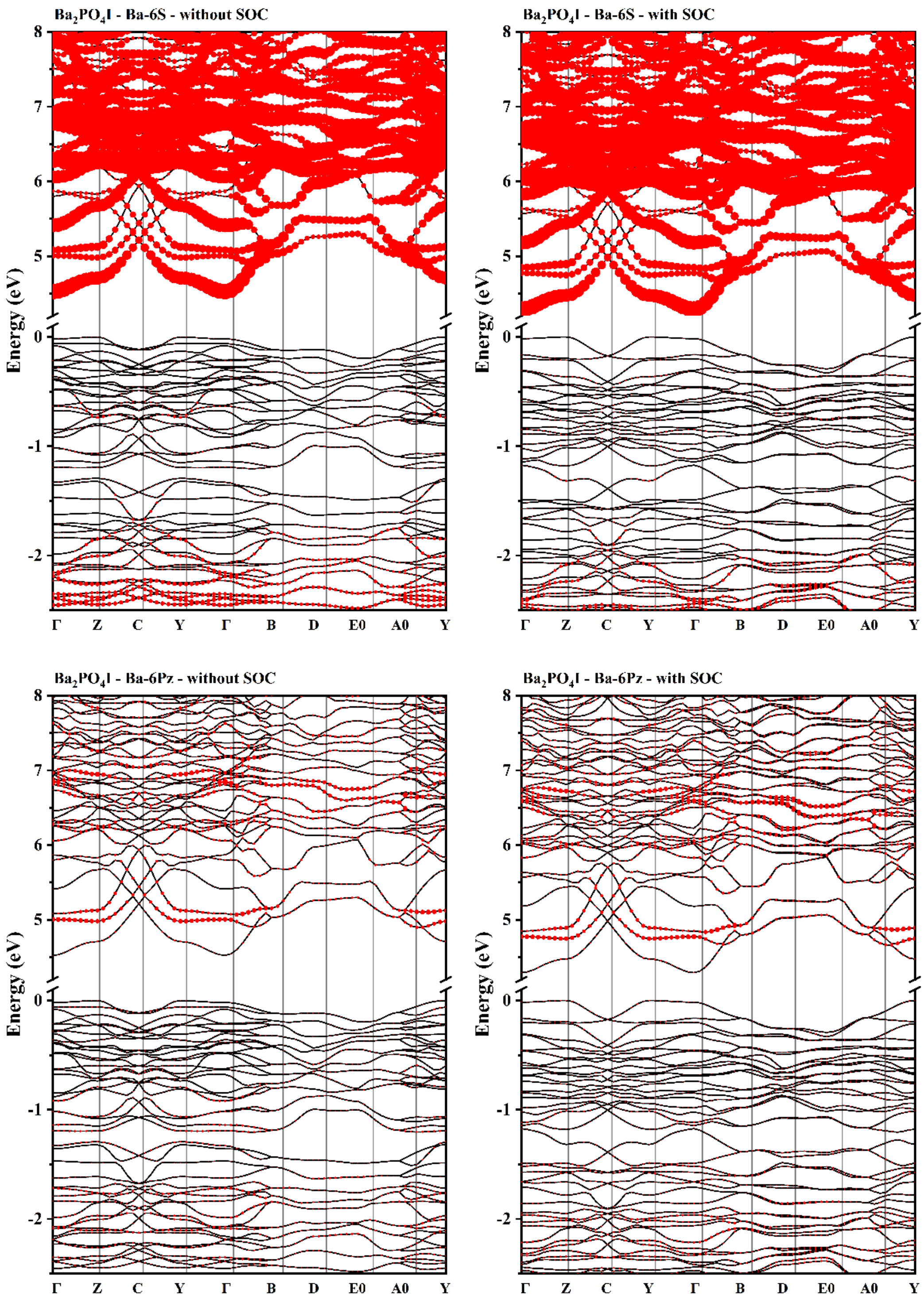

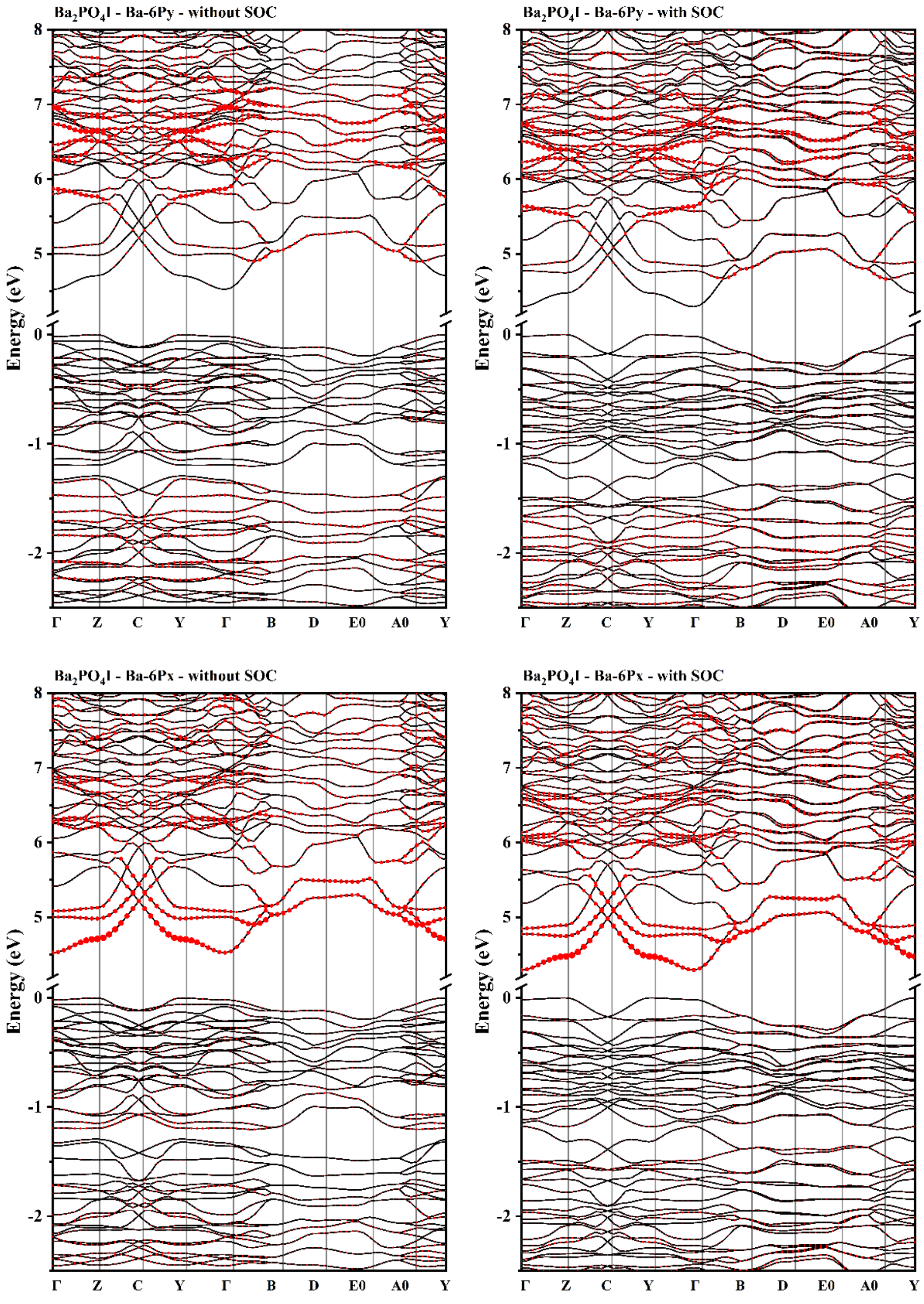

Ba_2PO_4I - Ba-6Py - without SOC
Ba_2PO_4I - Ba-6Py - with SOC
Ba_2PO_4I - Ba-6Px - without SOC
Ba_2PO_4I - Ba-6Px - with SOC
Energy (eV)
8
7
6
5
0
-1
-2
Γ
Z
C
Y
Γ
B
D
E0
A0
Y

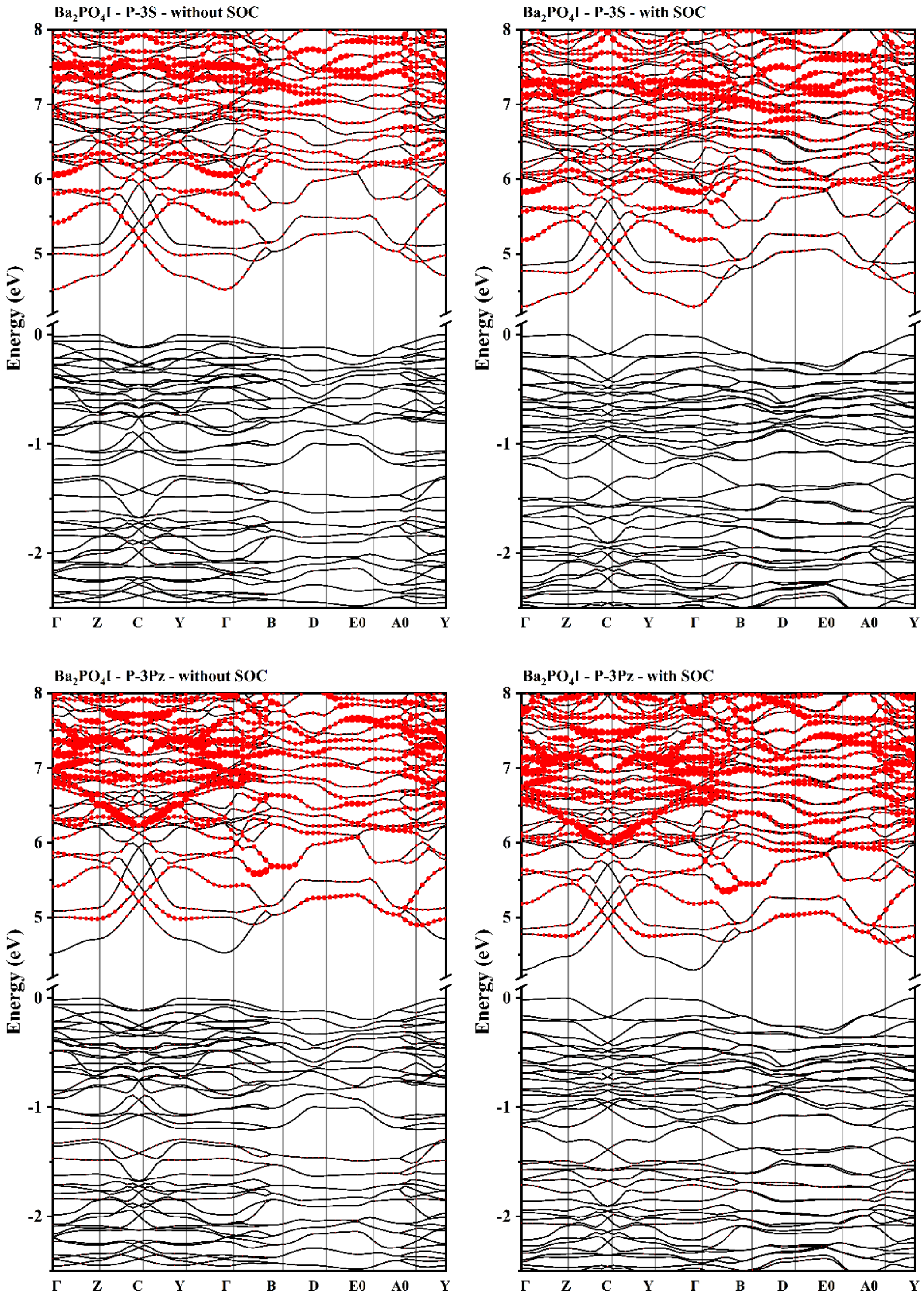

Ba_2PO_4I - P-3S - without SOC
Ba_2PO_4I - P-3S - with SOC
Ba_2PO_4I - P-3Pz - without SOC
Ba_2PO_4I - P-3Pz - with SOC
Energy (eV)
8
7
6
5
0
-1
-2
Γ
Z
C
Y
Γ
B
D
E0
A0
Y

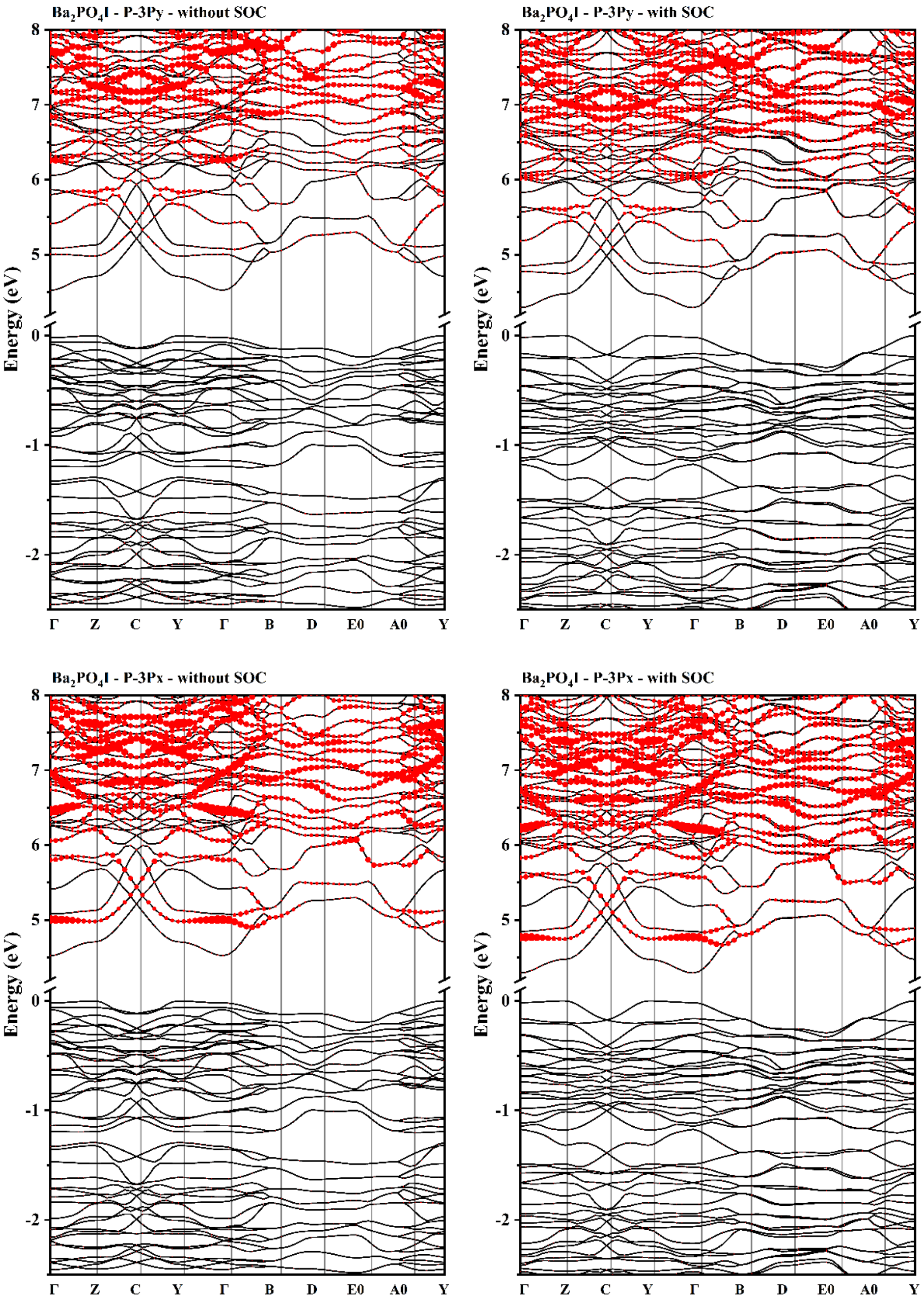
Ba_2PO_4I - P-3Py - without SOC
Ba_2PO_4I - P-3Py - with SOC
Ba_2PO_4I - P-3Px - without SOC
Ba_2PO_4I - P-3Px - with SOC
Energy (eV)
8
7
6
5
0
-1
-2
Γ
Z
C
Y
Γ
B
D
E0
A0
Y

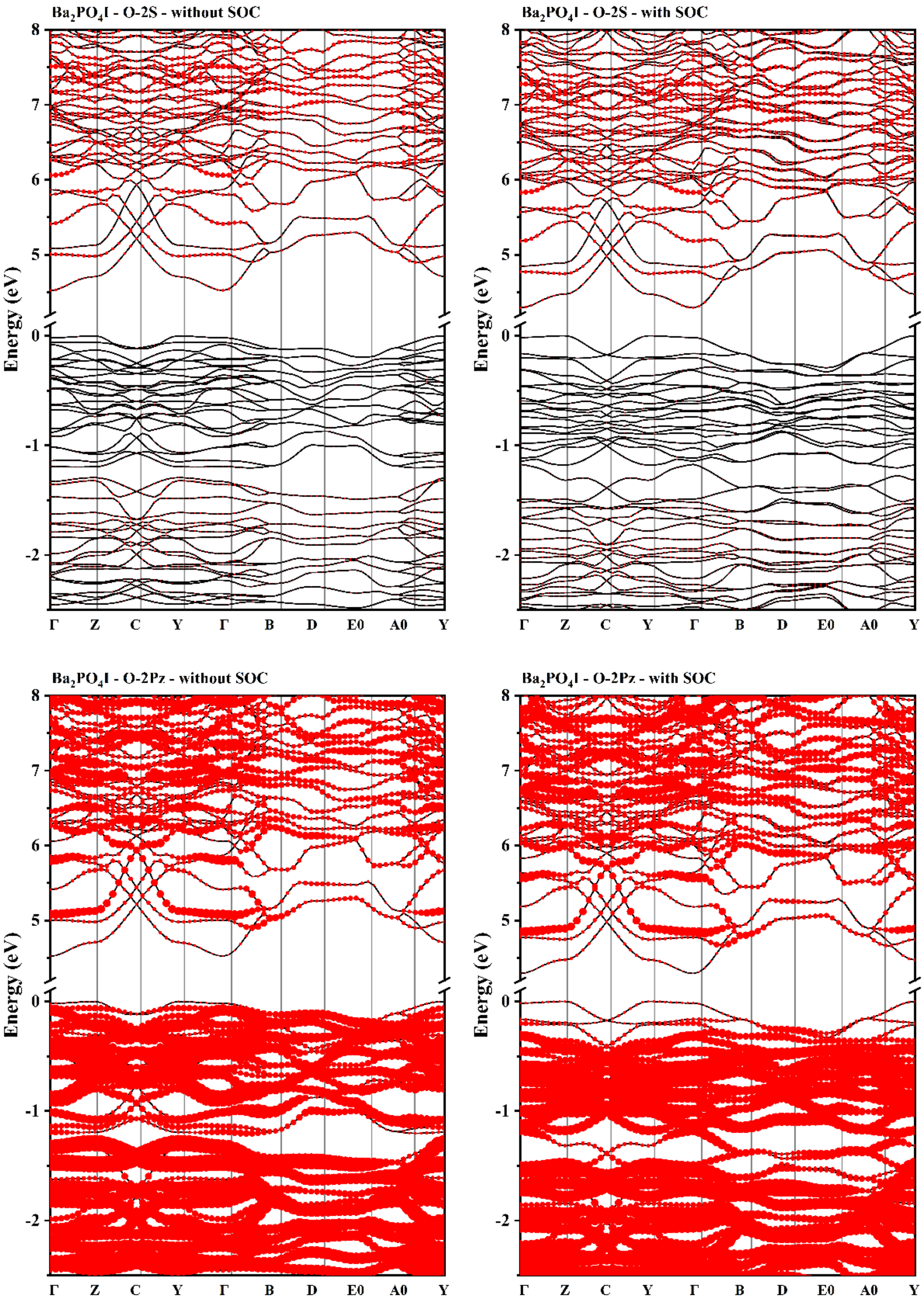

Ba2PO4I - O-2S - without SOC
Ba2PO4I - O-2S - with SOC
Ba2PO4I - O-2Pz - without SOC
Ba2PO4I - O-2Pz - with SOC
Energy (eV)
8
7
6
5
0
-1
-2
Γ Z C Y Γ B D E0 A0 Y

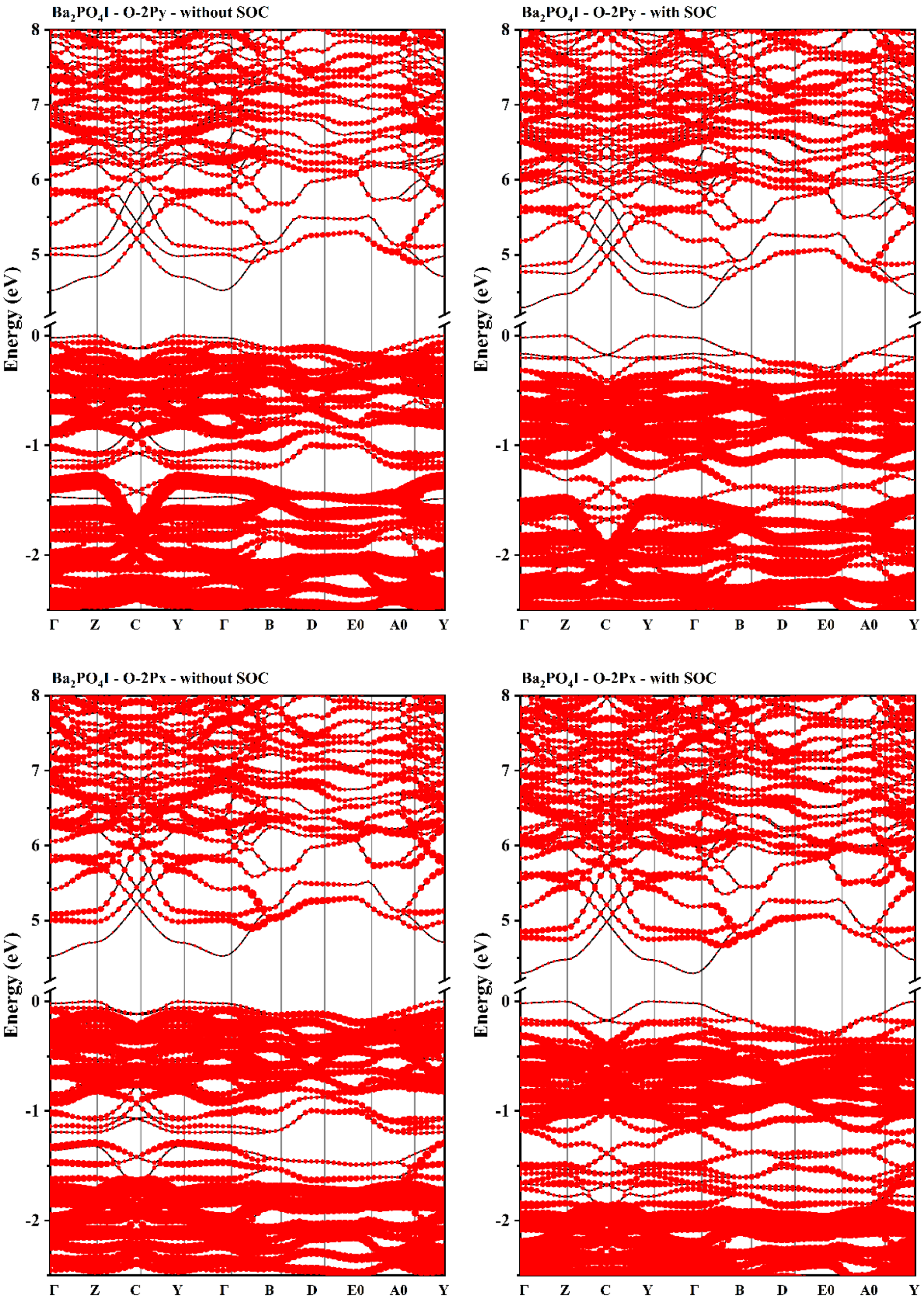

Ba2PO4I - O-2Py - without SOC
Ba2PO4I - O-2Py - with SOC
Ba2PO4I - O-2Px - without SOC
Ba2PO4I - O-2Px - with SOC
Energy (eV)
8
7
6
5
0
-1
-2
Γ Z C Y Γ B D E0 A0 Y

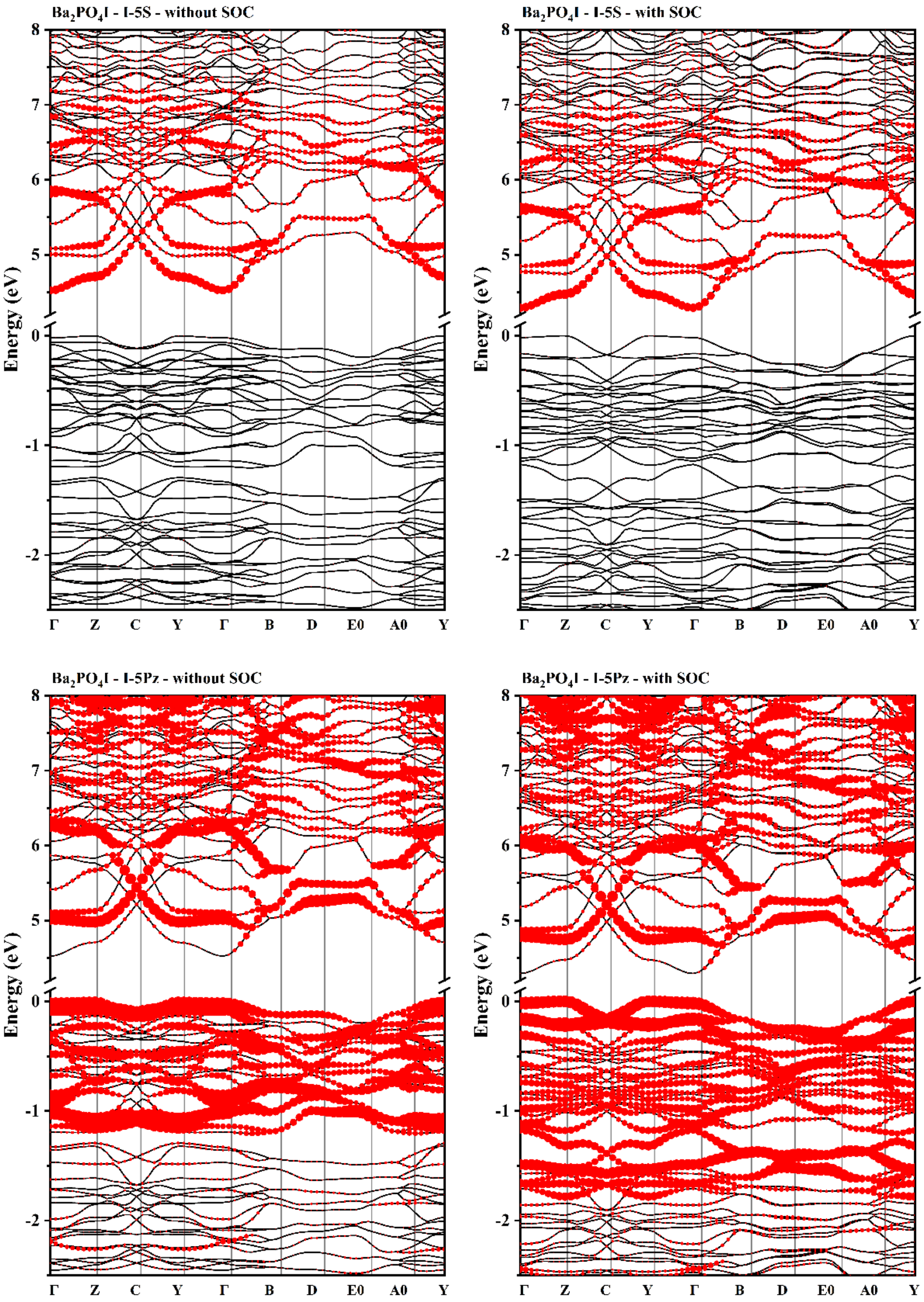

Ba2PO4I - I-5S - without SOC
Ba2PO4I - I-5S - with SOC
Ba2PO4I - I-5Pz - without SOC
Ba2PO4I - I-5Pz - with SOC
Energy (eV)
8
7
6
5
0
-1
-2
Γ Z C Y Γ B D E0 A0 Y

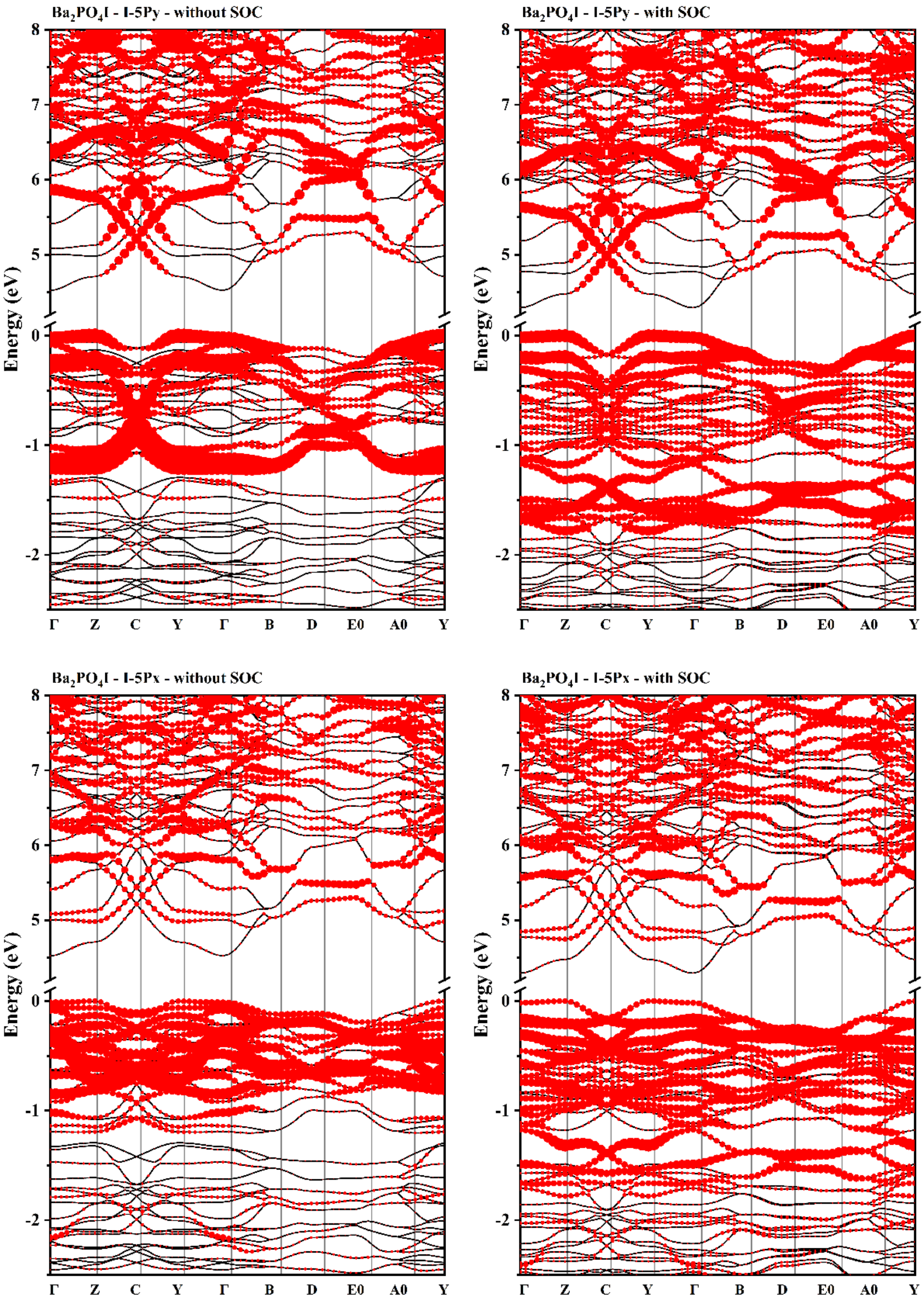
Ba_2PO_4I - I-5Py - without SOC
Ba_2PO_4I - I-5Py - with SOC
Ba_2PO_4I - I-5Px - without SOC
Ba_2PO_4I - I-5Px - with SOC
Energy (eV)
8
7
6
5
0
-1
-2
Γ Z C Y Γ B D E0 A0 Y

Figure S7. The projected band structure of Sr_2PO_4I along symmetry directions in the first Brillouin zone without (left panel) and with (right panel) spin-orbit effects.

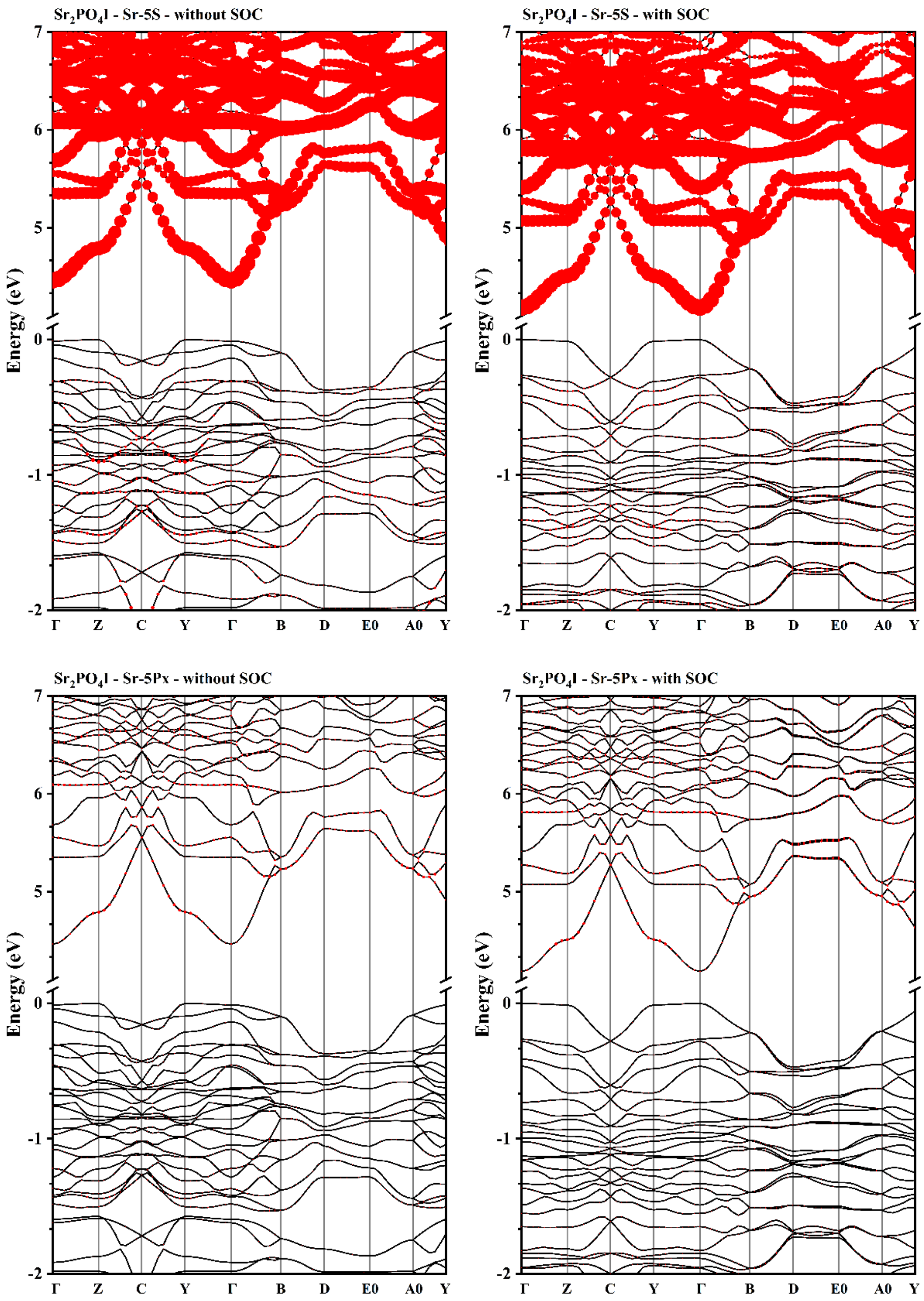

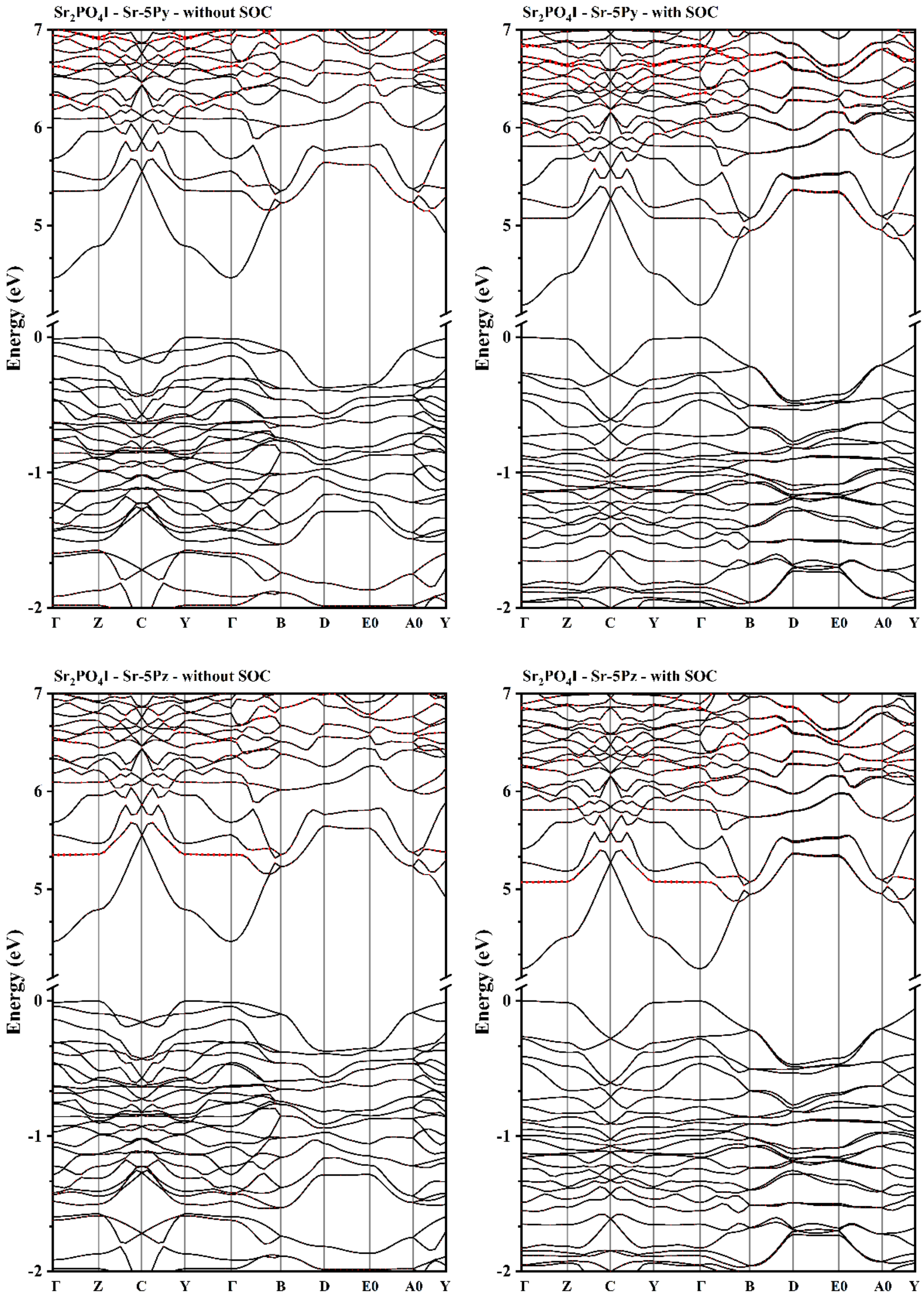

Sr2PO4I - Sr-5Py - without SOC
Sr2PO4I - Sr-5Py - with SOC
Sr2PO4I - Sr-5Pz - without SOC
Sr2PO4I - Sr-5Pz - with SOC
Energy (eV)
7
6
5
0
-1
-2
Γ Z C Y Γ B D E0 A0 Y

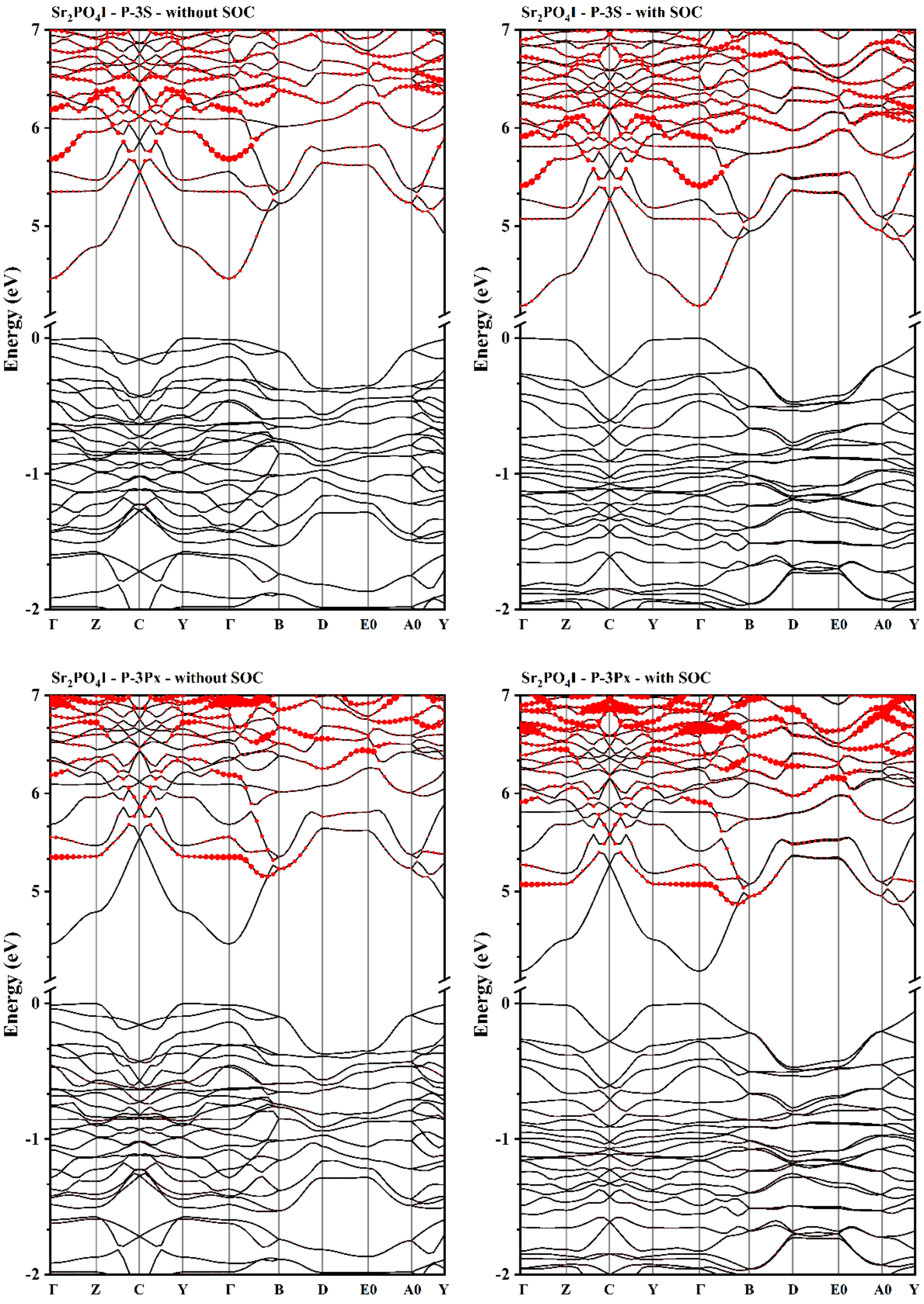
Sr2PO4I - P-3S - without SOC
Sr2PO4I - P-3S - with SOC
Sr2PO4I - P-3Px - without SOC
Sr2PO4I - P-3Px - with SOC
Energy (eV)
7
6
5
0
-1
-2
Γ Z C Y Γ B D E0 A0 Y

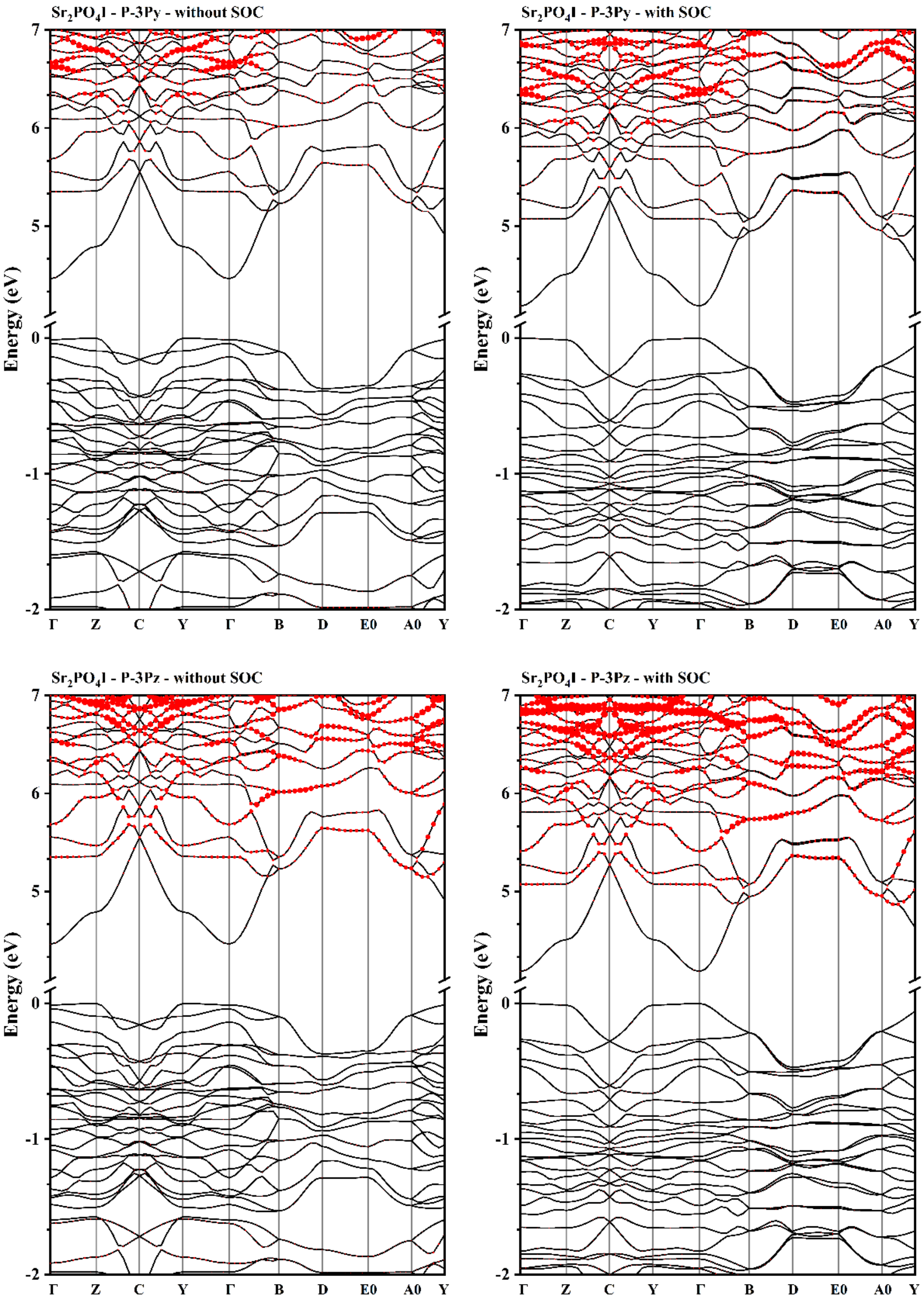

Sr_2PO_4I - P-3Py - without SOC
Sr_2PO_4I - P-3Py - with SOC
Sr_2PO_4I - P-3Pz - without SOC
Sr_2PO_4I - P-3Pz - with SOC
Energy (eV)
7
6
5
0
-1
-2
Γ Z C Y Γ B D E0 A0 Y

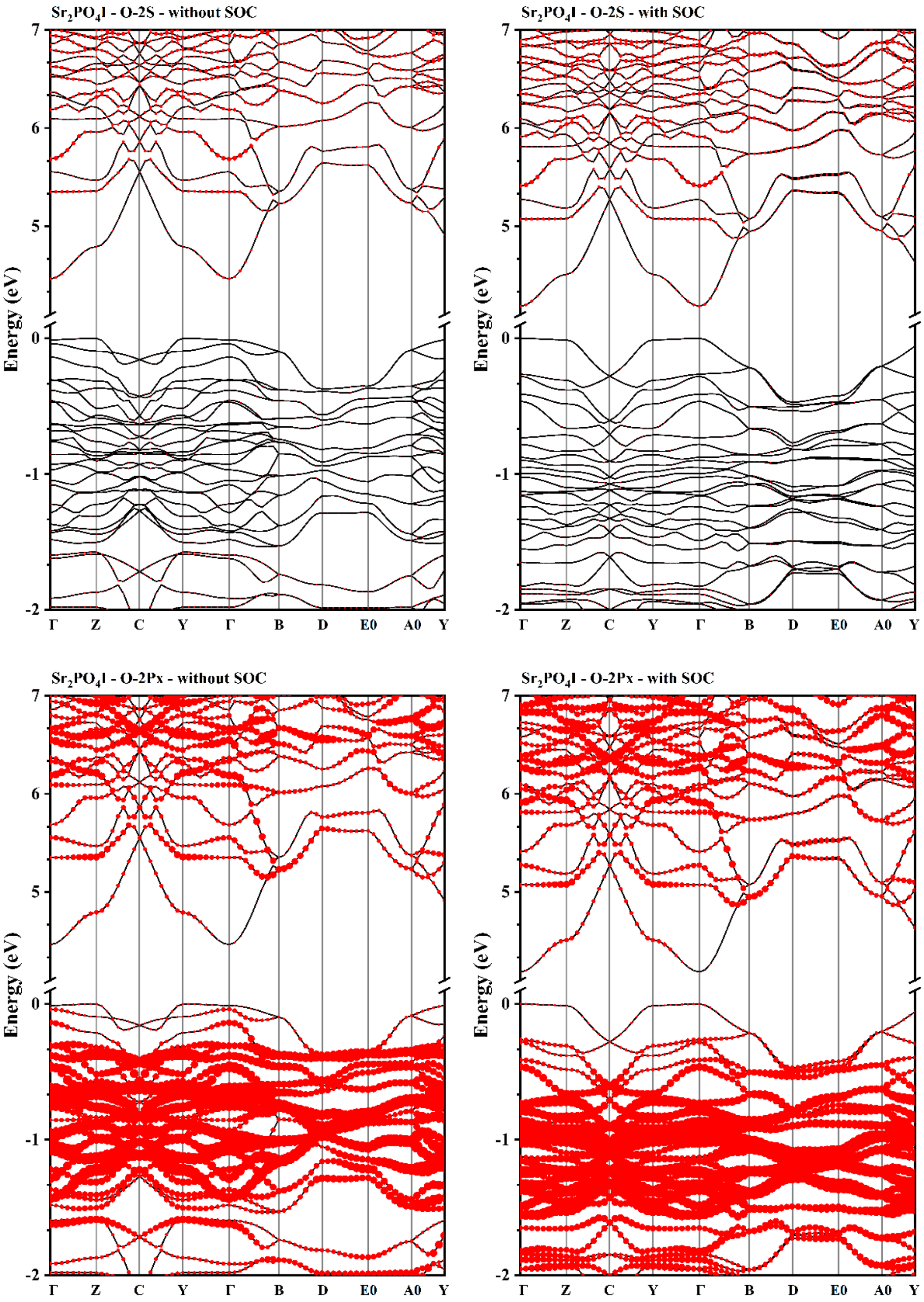
Sr2PO4I - O-2S - without SOC
Sr2PO4I - O-2S - with SOC
Sr2PO4I - O-2Px - without SOC
Sr2PO4I - O-2Px - with SOC
Energy (eV)
7
6
5
0
-1
-2
Γ Z C Y Γ B D E0 A0 Y

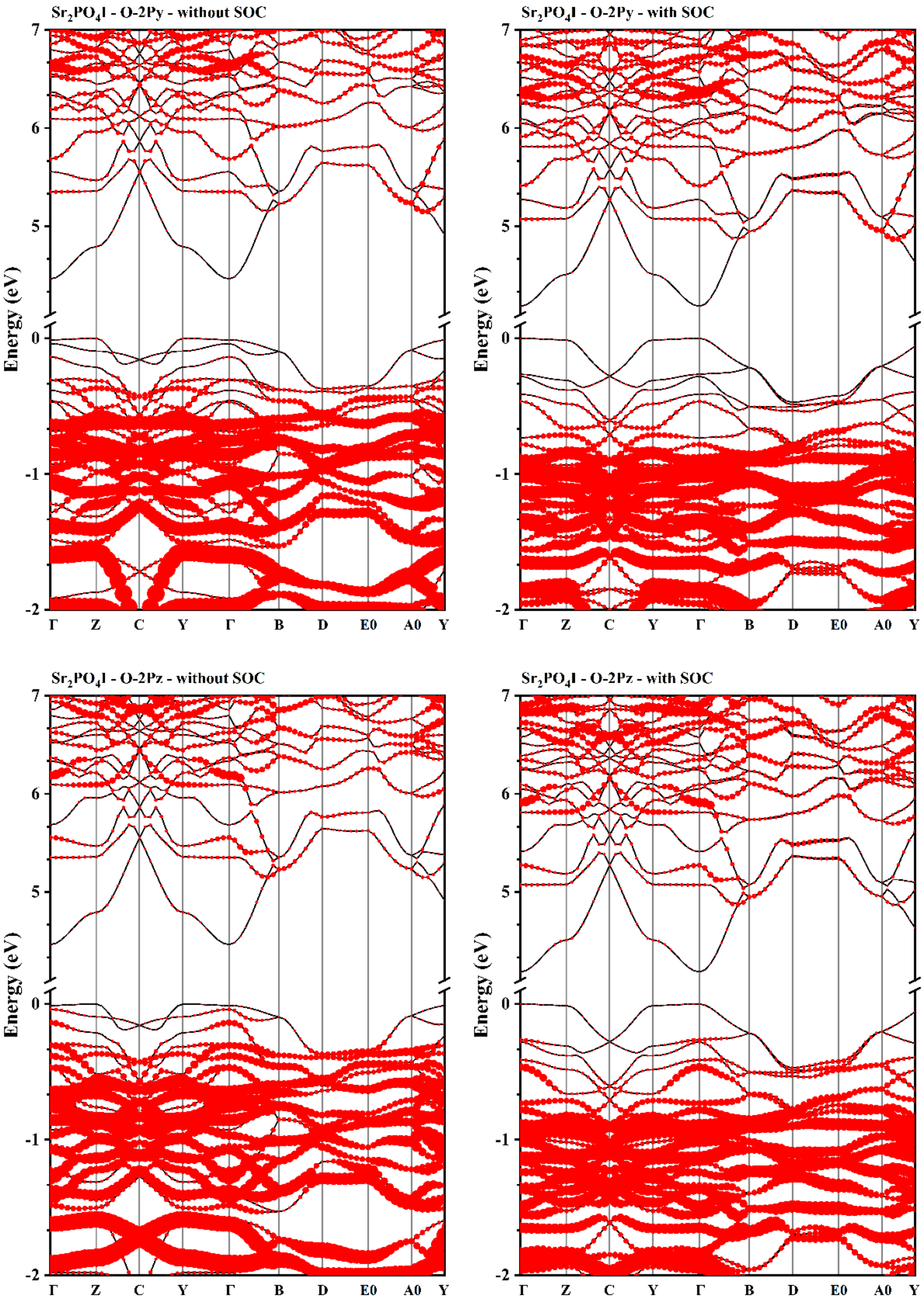
Sr2PO4I - O-2Py - without SOC
Sr2PO4I - O-2Py - with SOC
Sr2PO4I - O-2Pz - without SOC
Sr2PO4I - O-2Pz - with SOC
Energy (eV)
7
6
5
0
-1
-2
Γ Z C Y Γ B D E0 A0 Y

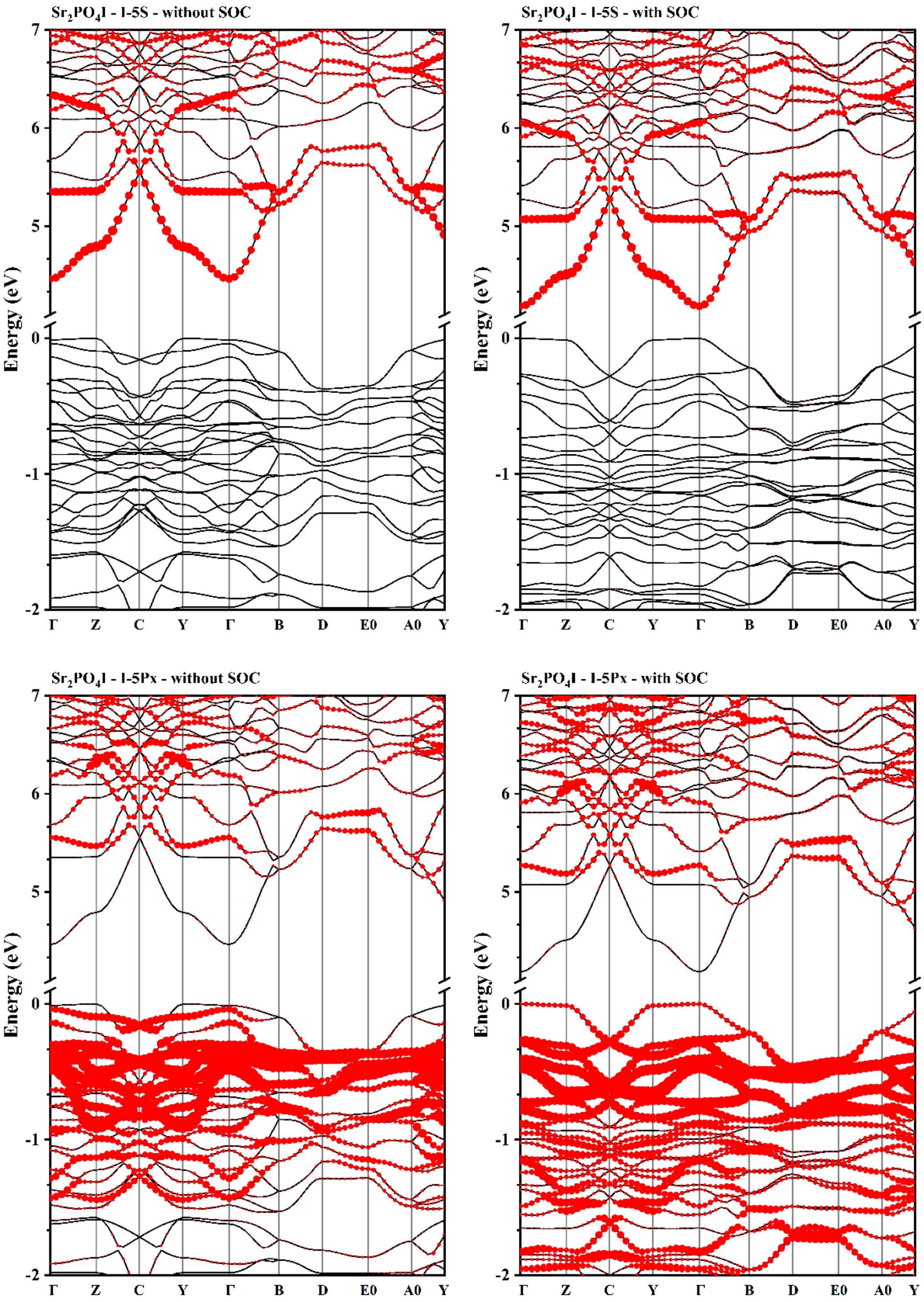

Sr_2PO_4I - I-5S - without SOC
Sr_2PO_4I - I-5S - with SOC
Sr_2PO_4I - I-5Px - without SOC
Sr_2PO_4I - I-5Px - with SOC
Energy (eV)
7
6
5
0
-1
-2
Γ Z C Y Γ B D E0 A0 Y

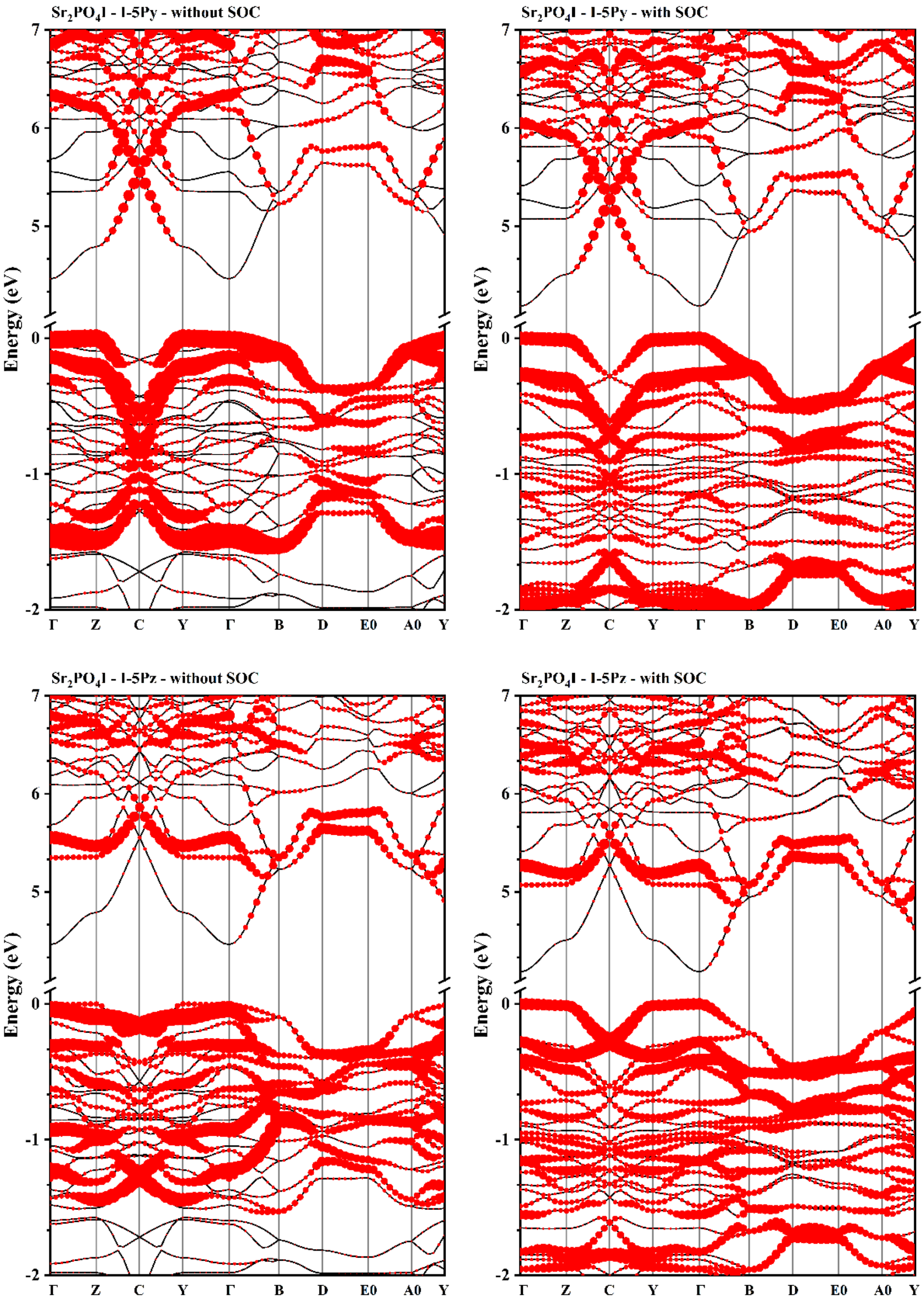
Sr2PO4I - I-5Py - without SOC
Sr2PO4I - I-5Py - with SOC
Sr2PO4I - I-5Pz - without SOC
Sr2PO4I - I-5Pz - with SOC
Energy (eV)
Γ Z C Y Γ B D E0 A0 Y
